# Novel Approaches for the Treatment of Alzheimer’s and Parkinson’s Disease

**DOI:** 10.3390/ijms20030719

**Published:** 2019-02-08

**Authors:** Michiel Van Bulck, Ana Sierra-Magro, Jesus Alarcon-Gil, Ana Perez-Castillo, Jose A. Morales-Garcia

**Affiliations:** 1 Instituto de Investigaciones Biomédicas (CSIC-UAM), Arturo Duperier, 4. 28029 Madrid, Spain; mvbulck@iib.uam.es (M.V.B.); anasierra@iib.uam.es (A.S.-M.); jalarcon@iib.uam.es (J.A.-G.); aperez@iib.uam.es (A.P.-C.); 2 Centro de Investigación Biomédica en Red sobre Enfermedades Neurodegenerativas (CIBERNED), Valderrebollo, 5, 28031 Madrid, Spain; 3 Departamento de Biología Celular, Facultad de Medicina, Universidad Complutense de Madrid (UCM), Plaza Ramón y Cajal s/n, 28040 Madrid, Spain

**Keywords:** aging, Alzheimer, Parkinson, neurodegeneration, neuroinflammation, neurogenesis, novel approaches

## Abstract

Neurodegenerative disorders affect around one billion people worldwide. They can arise from a combination of genomic, epigenomic, metabolic, and environmental factors. Aging is the leading risk factor for most chronic illnesses of old age, including Alzheimer’s and Parkinson’s diseases. A progressive neurodegenerative process and neuroinflammation occur, and no current therapies can prevent, slow, or halt disease progression. To date, no novel disease-modifying therapies have been shown to provide significant benefit for patients who suffer from these devastating disorders. Therefore, early diagnosis and the discovery of new targets and novel therapies are of upmost importance. Neurodegenerative diseases, like in other age-related disorders, the progression of pathology begins many years before the onset of symptoms. Many efforts in this field have led to the conclusion that exits some similar events among these diseases that can explain why the aging brain is so vulnerable to suffer neurodegenerative diseases. This article reviews the current knowledge about these diseases by summarizing the most common features of major neurodegenerative disorders, their causes and consequences, and the proposed novel therapeutic approaches.

## 1. Introduction

Neurodegenerative diseases are incurable and debilitating conditions that result in progressive degeneration and/or death of nerve cells. The huge variety of neurodegenerative diseases, in terms of pathological characteristics, symptoms, and treatments, makes it very difficult to classify them in general terms. Taking into account the different neurodegenerative diseases, one of the parameters we can considerer is prevalence. According to this, there are two most prevalent neurodegenerative diseases: Alzheimer’s disease (AD) and Parkinson’s disease (PD) [1,2]. This affirmation is substantiated in the literature. In 2015, there were 46.8 million AD patients worldwide with direct and indirect costs to society of 81,800 million USD [2], and in 2016 there were 6.1 million individuals with PD worldwide [1]. Dementia is a major symptom of AD, consisting of memory impairment accompanied by dysfunction, which is responsible for the inability to develop daily life activities [3]. Vascular dementia is quite important since it is considered the second most common cause of dementia in the aging population and also is thought to underlie AD. This disorder consists of a decline in cognitive skills caused by blocking or reduction of blood flow to the brain [4]. On the other hand, PD is characterized by motor symptoms such as bradykinesia/akinesia, resting tremor, rigidity, and postural abnormalities, and non-motor symptoms such as dementia, hyposmia, depression, and emotional changes [5].

Behavioral and psychological symptoms of dementia (BPSD) represent neuropsychiatric symptoms occurring in subjects with dementia. They are clinically relevant as cognitive symptoms as they strongly correlate with the degree of functional and cognitive impairment. BPSD include anxiety, elation, hallucinations, agitation, irritability, abnormal motor behavior, apathy, depression, and sleep or appetite changes [6]. Although some of these BPSDs could be alleviated by the use of atypical antipsychotics, these compounds have been described to induce clinically significant metabolic adverse events, increasing mortality in patients with dementia [7,8].

Despite the high prevalence of these diseases, the treatments available today are not able to significantly modify the progress of the disease, since these treatments only treat the symptoms of it. A substantial part of traditional treatments for neurodegenerative diseases including AD, cerebral amyloid angiopathy, frontotemporal dementia, mild cognitive impairment, and PD are based on immunotherapy, most of them active and passive immunotherapy trials conducted based on amyloid, tau, and α-synuclein targeting (Alzforum.org; https://www.alzforum.org/therapeutics). Some other studies have suggested the use of small molecules for AD treatment, able to cross the BBB, like statins. These include simvastatin, a HMG-CoA reductase inhibitor, approved by FDA for the treatment of hypercholesterolemia and diabetic cardiomyopathy. In that sense retrospective clinical studies directed by Wolozin and colleagues, demonstrated a significant reduction (at least 50%) in the risk incidence of suffering AD and PD after simvastatin administration [9,10]. Now, research is focusing on finding new disease-modifying therapies to slow disease progression [11,12].

Better understanding of the triggering factors involved in the onset and progression of the disorders are crucial for developing novel therapies. The main characteristic of these diseases is the existence of two subtypes: Familial and idiopathic forms. The familial form, which an average of 5% of patients will develop before 65 years of age, is associated with various genetic mutations. The idiopathic form of the disease, which accounts for 95% of cases, is associated with aging and has some other unrevealed risk factors [2,13]. In the case of AD, the familial form is caused by mutations of genes coding for amyloid precursor protein (*APP*), presenilin-1 (*PSEN-1*), and presenilin-2 (*PSEN-2*) [2]. In familial PD, there is a longer list of related gene mutations, for example, α-synuclein (*SNCA*), Leucine-rich repeat kinase 2 (*LRRK2*), PTEN induced kinase 1 (*PINK1*), ATPase cation transporting 13A2 (*ATP13A2*), and other genes, like *PARK* [13]. The importance of identifying and understanding the roles of these genes in the underlining pathological mechanism could reveal new therapeutic approaches.

The presence of common pathological pathways involved in various neurodegenerative diseases that are associated with genes mutations that provoke familial forms could be promising approaches for treatments in AD and PD [14]. At the same time, different etiologies share similar underlying pathological pathways. Unfortunately, this is not a coincidence, since mutations at different steps of the same cellular processes, in the case of familiar disease and aging, share common risk factors. Understanding the relations and distinctions between these pathological processes at the molecular, cellular, and physiological levels need to be described carefully. One example of this concept can be explained with the gene coding for the triggering receptor expressed on myeloid cells 2 (TREM2) and genetic risk factors in different neurodegenerative diseases [15]. The TREM2 mutation exacerbates dysfunction in molecular receptor-mediated pathways related to inflammation, which downregulates good cellular responses, which provokes dysregulation of immune responses in the brain [15]. This example indicates how a pathological process can be studied and used as a targeting approach at different biological levels.

In general, the most important pathological processes that underline these diseases are: misfolding proteins and protein aggregates, mitochondria dysfunction, oxidative stress, ER stress, autophagy impairment, alteration of intracellular calcium homeostasis, inflammation, and neurogenesis impairment [14,16,17,18,19,20]. We know that there are similar pathological processes involved at different biological levels that might provoke neurodegeneration. The attempt to search for new targets involved in neurodegeneration could bring us closer to disease-modifying drug compounds. For this reason, the aim of this review is to summarize the new research strategies that are being used to identify disease-modifying therapies through the knowledge of the common pathological process that underlines neurodegenerative diseases. Here, we focus on two of the most prevalent neurodegenerative diseases: Parkinson’s disease and Alzheimer’s disease.

## 2. Pathological Targets in Neurodegenerative Diseases

**Figure 1:**
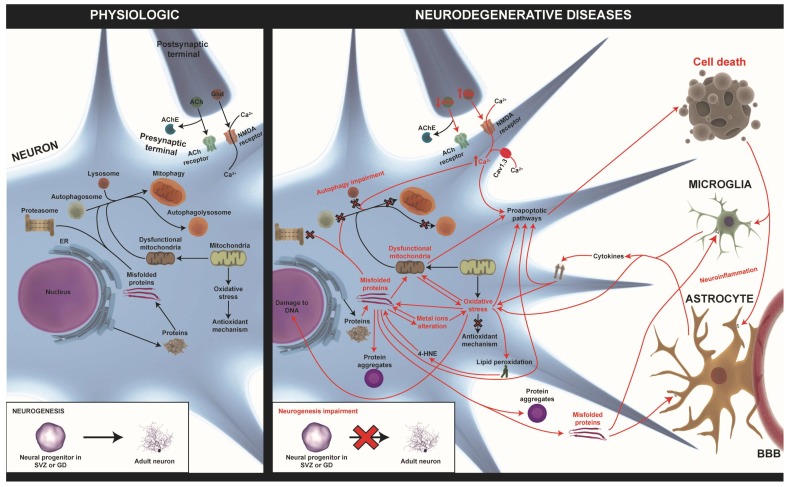
The general pathways involved in neurodegenerative diseases. Physiological processes like endosomal-lysosomal autophagy, neuroinflammatory responses, mitochondrial homeostasis, proteostasis, and metabolic profiling (proteome and lipidome) are dysregulated in neurodegenerative diseases (red arrows). Alterations in homeostasis mechanisms like the endosomal–proteosomal–autophagy pathway and an increase in misfolded protein aggregation are major factors in Alzheimer’s disease (AD) and Parkinson’s disease (PD). The oxidative stress caused by mitochondrial dysfunction and dysregulation of endogenous antioxidant mechanisms is influenced by the level of free radicals. The positive feedback loop between oxidative stress, misfolded proteins, and mitochondrial dysfunction is crucial in therapeutic interventions. Furthermore, pre- and post-synaptic integrity loss due to alterations in calcium homeostasis together with the above pathways is an important mechanism involved in proapoptotic pathway activation. In addition, the remains of dead cells and the misfolded proteins released into the extracellular environment provoke glia-activation, which releases cytokines and free radicals, exacerbating neuronal death, which establishes another negative feedback loop between neurodegeneration and neuroinflammation. Finally, these alterations also affect neurogenesis in Alzheimer’s disease (AD) and Parkinson’s disease (PD).

To understand how these disease-modifying therapies act, we need to know the pathological events that characterize these diseases (Figure 1).

### 2.1. Misfolded Proteins and Protein Aggregates

Most neurodegenerative diseases are characterized by protein aggregates formed mainly by a specific protein that varies in each disease. In general, all these proteins are characterized by very large disoriented domains without a defined secondary structure. When these proteins are prone to aggregation (oligomers to fibrils) in pathology, rich β-sheets are promptly available as secondary structures [21]. In the case of AD, two types of protein aggregate appear: soluble intracellular (monomer to oligomers) aggregates and insoluble extracellular (proto-fibril to fibrils) aggregates, which are mainly formed by beta amyloid (Aβ) [22], and other intracellular aggregates called neurofibrillary tangles (NFT) formed by hyperphosphorylated tau protein (tau) [23]. In PD, an intracellular protein aggregate named the Lewy Body appears, which is formed by misfolded α-synuclein (α-Syn) proteins [24].

The causes of neurofibrillary tangles and Lewy Body formation are unknown. However, there are several hypotheses, including oxidative stress and mitochondrial dysfunction, for their starting point [25,26]. In the case of Aβ plaques, which involve conformational and aggregate changes, the causes are unclear. Besides this, possible pathways have been discovered for Aβ formation. Aβ is formed by the differential processing of amyloid precursor protein (APP). APP can be processed by the α-secretase enzyme, giving rise to the soluble amyloid precursor protein-α (sAPPα) fragment, which follows the “physiological” non-amyloidogenic pathway or by the enzyme β-secretase (BACE1), prompting the soluble amyloid precursor protein-β (sAPPβ) fragment, which follows the “pathophysiological” amyloidogenic pathway. Finally, both types of fragment are processed by γ-secretase, where sAPPα does not result in molecules with pathogenic potential, while sAPPβ gives rise to the Aβ peptide species, the main component of Aβ plaques [27].

Despite this, cells have physiological mechanisms to maintain proteostasis and degradation of protein aggregates, such as macroautophagy, chaperone-mediated autophagy, or the ubiquitin-proteasome system which appears to be altered in AD and PD. However, many of these aggregates and proteins are partially resistant to degradation by these mechanisms, progressively losing their effectiveness due to the sequestration of chaperones by the aggregates [21,27]. In addition, the appearance of misfolded proteins and aggregates that cannot be degraded will follow a prion-like propagation mechanism involving misfolding protein aggregates between cells [21].

The importance of these misfolded proteins and aggregates lies in their cytotoxic capacity through different mechanisms. However, it should be noted that only the oligomers and fibrils that precede the formation of the aggregates are toxic; therefore, the aggregates are proposed to be a protective mechanism against toxic forms [28]. Thus, the cytotoxic effects of these misfolded proteins, apart from the effects on the machinery for proteostasis [21], are mitochondrial damage, ER stress, autophagy, and calcium homeostasis impairment [17,27].

### 2.2. Mitochondrial Dysfunction

Nowadays, it is well known that mitochondria are involved in several important cell functions besides ATP generation, such as the maintenance of calcium (Ca^2+^) homeostasis, intrinsic apoptosis activation through cytochrome *c* release, modification of the reduction-oxidation potential of cells, and oxidative stress regulation [29]. However, mitochondria can also contribute to neuronal death in the context of age-related neurodegenerative disorders through a number of mechanisms including the alteration of mitochondrial dynamics (fission/fusion and organelle trafficking) and biogenesis/mitophagy processes; impairment of Ca^2+^ homeostasis; mutations on mitochondrial DNA (mtDNA); incorrect activation of apoptosis; oxidative stress; and alteration of cellular metabolism [30]. These mitochondria malfunctions can be due to different reasons. For example, as mentioned above, misfolded proteins are a common feature in neurodegenerative diseases, and there are a number of ways in which these proteins can alter mitochondrial activity, such as their direct association with mitochondrial structures, damage to mtDNA, impairment to organelle trafficking and dynamics, deregulation of bioenergetics and quality control pathways, and stimulation of mitochondria-dependent cell death [31]. On the other hand, some evidence suggests that the accumulation of α-Syn in PD and phospho-tau and Aβ in AD may be direct consequences of mitochondrial dysfunction [32,33]. Also, mutations of some nuclear genes that occur in neurodegenerative disorders affect proteins involved in mitochondrial activity which can trigger mitochondrial dysfunction in such pathologies. For instance, PD-related mutations in phosphatase and tensin homolog (PTEN)-induced putative kinase 1 (PINK1) and Parkin proteins have been implicated in mitochondrial quality control [34] and elicit mitophagy decrease and mitochondrial dysfunction [35,36,37]. Another mutation that occurs in some PD cases is in DJ1 protein, a chaperone involved in protection from oxidative stress [38]. This produces increased sensitivity to this stress in cells, impairs mitochondrial respiratory chain complexes, decreases ATP production, and alters mitochondrial morphology [39,40]. Similarly, some AD related mutations can alter mitochondrial activity. This is the case for PSEN-1 and PSEN-2 proteins which are localized on the endoplasmic reticulum (ER) mitochondrial associated membranes (MAMs), and their mutation triggers increased cytosolic Ca^2+^ levels in these regions, inducing mitochondrial Ca^2+^ overload and stimulation of mitochondrial respiration, thereby increasing reactive oxygen species (ROS) generation [41].

### 2.3. Oxidative Stress

The intracellular balance between oxidants and antioxidants is regulated by the production of free radicals by the mitochondria (electron transport chain and different enzymes), the ER, peroxisomes, or different enzymes (e.g., NAPDH oxidases or xanthine oxidases), and the reduction of these free radicals by different antioxidant mechanisms, such as glutathione, superoxide dismutase, catalase, and peroxiredoxins. However, in neurodegenerative diseases, this balance is broken and there is a situation of oxidative stress, mainly due to the mitochondrial dysfunction that occurs in these diseases, which causes a high increase in the production of free radicals, and the cellular antioxidant mechanisms cannot confront it [42]. In addition, in the case of AD, there are several pathological mechanisms that cause an increase in oxidative stress, such as the activation of NADPH oxidases by Aβ peptides with the consequent production of free radicals by this enzyme, the overactivation of *N*-methyl-d-aspartate receptors (NMDAR) (excitotoxicity) that promotes an influx of Ca^2+^ to the cell, causing an increase in the production of free radicals [43], or the binding of the Aβ peptide to metals, which leads to the production of free radicals [42,44]. In the case of PD, oxidative stress also comes from auto-oxidation of dopamine that, when free in the cytoplasm of dopaminergic (DA) neurons [45], provokes the constant flow of Ca^2+^ in these neurons, which causes them to have a basal firing rate, but also makes them more susceptible to oxidative stress [46,47]. The high energy demand suffered by this type of neuron also triggers higher production of free radicals due to greater mitochondrial respiratory function [45,47]. In both cases, we must bear in mind that the neuroinflammation present in these diseases also aggravates oxidative stress [14].

The problem with oxidative stress is the intracellular cytotoxic consequences that mainly derive from the modification of molecules due to the free radicals—highly reactive molecules that alter all the molecules they find in their case [42]. Thus, at a molecular level, this oxidative stress causes the peroxidation of lipids and formation of 4-hydroxynonenal (4-HNE), which can react with proteins to generate adducts or alter their conformation; carbonylation and nitration of proteins with the consequent loss of their conformation; and oxidation of nitrogenous bases, which leads to mutations and destabilization of nucleic acids [48]. These biochemical alterations to certain biomolecules trigger high consequences at the cellular level such as alteration of the integrity of the membranes; loss of conformation of numerous proteins, which can cause, among other problems, alterations to metabolism or alterations in the mechanisms of mutation correction in the DNA; a decrease in ATP levels; alterations in mitochondrial function, such as high production of free radicals by the electron transport chain, which establishes a positive feedback loop for the production of free radicals; and an increase in the misfolding of the Aβ, tau, and α-Syn proteins [48,49]. Finally, due to the action of all these cytotoxic processes in the different compartments of the cell, oxidative stress is able to activate proapoptotic pathways that lead to the death of neurons, including the intrinsic pathway of apoptosis, due to damage to the mitochondria [50].

### 2.4. Autophagy Impairment

Autophagy is a cellular mechanism that carries out the degradation and recycling of different cellular components, including the degradation of misfolded aggregates and proteins. However, we can differentiate three different ways in which it is performed: Chaperone-dependent autophagy, microautophagy, and macroautophagy [51]. The first process is carried out through the ubiquitination of the misfolded proteins and their subsequent treatment by chaperones. However, as previously commented, when the function of the chaperones in these diseases is altered, this option cannot be performed [52]. On the other hand, the other two autophagy methods are also altered in most neurodegenerative diseases for various reasons that can be related to all the steps necessary to carry them out, including the biogenesis of autophagosomes or lysosomal function. An example of the relationship between autophagy and neurodegenerative diseases is the fact that numerous genetic risk factors for them are related to autophagy, as is the case of *ATP13A2* and *VPS35* for PD or the case of *PICALM* and *PSEN-1* for AD [53].

Thus, as indicated, a primary function of autophagy is the degradation of misfolded proteins and aggregates. However, this is not the only function of autophagy related to neurodegenerative diseases, since, for example, autophagy has also been considered an antioxidant mechanism [54] or, specifically, because macro-autophagy is also responsible for the degradation of dysfunctional mitochondria (mitophagy), [54,55]. Therefore, the alteration of the macroautophagy present in these diseases can trigger a poor withdrawal of the dysfunctional mitochondria present in the cell, which exacerbates the negative consequences of mitochondrial dysfunction [54,55]. In addition, in the case of PD, mutations in the *PINK1* gene, a genetic risk factor for this disease, are related to one of the main pathways of mitophagy [55].

### 2.5. Intracellular Ca^2+^ Homeostasis Alteration

The intracellular homeostasis of calcium is extremely important in the neural cells present in the Central Nervous System (CNS) because it acts as a second messenger in many pathways, such as the release of neurotransmitters by neurons, synaptic plasticity, astrocyte Ca^2+^ waves, activation of proapoptotic enzymes, etc. [56]. That is why the concentration of this ion is highly controlled, being the main storage center of this ion in the ER [57]. However, Ca^2+^ homeostasis is altered in neurodegenerative diseases, which leads to strong cytotoxic and dysfunctional consequences for neurons [17], which usually lead to alterations in the functions of enzymes regulated by Ca^2+^ concentrations, thereby provoking an increase in protein aggregation; alteration of lysosomal function and, therefore, autophagic function [57]; a decrease in long-term potentiation (LTP); and increase in long-term depression (LTD) [56], among others.

Nowadays, there are several compatible hypotheses to explain the dysregulation of the Ca^2+^ concentration that occurs in neurodegenerative diseases, including the possibility that it is due to the effects of other cellular alterations present in these diseases. One of these hypotheses is that it is because of the process of excitotoxicity, which occurs on account of an overactivation of glutamate NMDAR, which is due to different reasons depending on the pathology, but, in general, provokes an overload of Ca^2+^ inside the cell, which has cytotoxic consequences for the neuron. For example, this can cause the activation of proapoptotic enzymes such as calpains [17]. In addition, in the case of AD, some of the hypotheses that explain this dysregulation are the ability of the Aβ peptide to cause pores in the ER that release Ca^2+^ from its interior to the cytoplasm or the effect of mutations in presenilins that provoke this alteration in Ca^2+^ concentration [56].

### 2.6. Neuroinflammation

Glial cells (astrocytes and microglia) have been recognized to have a clear function in neurodegenerative diseases since past decades. However, these cells are extremely important because they have immune functions, which, under physiological conditions, result in controlled reactions, but, under pathophysiological conditions, such as neuronal death, give rise to exacerbated reactions in the midterm. In this way, in neurodegenerative diseases, a positive feedback loop is created between neuronal death and neuroinflammation [14].

The reasons for this neuroinflammation are diverse, such as the activation of astrocytes and microglia by the binding of the misfolded proteins of each disease to the toll-like receptors (TLR) of these cells [58] and the activation of other damage-associated molecular pattern (DAMPS) receptors of these cells by the damage signals present in these diseases. The exposure of these cells to misfolded proteins and to cell damage signals in chronic diseases leads to an exacerbated immune reaction by the glia cells. In this way, this reaction provokes the chronic production of free radicals and pro-inflammatory cytokines that activates neuronal death pathways, in addition to activating other astrocytes and microglia cells [59].

### 2.7. Neurogenesis Impairment

Adult neurogenesis in humans is a mechanism of cerebral plasticity and, therefore, is fundamental for the correct functioning of the nervous system. Although several years ago it was believed that this neurogenesis process only occurred at the early stages, many studies so far have shown it to take place in adults, specifically in the subventricular zone and the subgranular cells of the dentate gyrus of the hippocampus [60]. In some neurodegenerative diseases, it is clearly the pathogenic decrease of neurogenesis, but the reason why it happens is unclear and depends on the disease. Nonetheless, adult neurogenesis is generally affected by neuroinflammation, which causes a decrease in this process [61,62].

It has been shown that impaired neurogenesis is associated with Alzheimer’s and Parkinson’s diseases. Firstly, this is because adult neurogenesis in humans is necessary to maintain certain cognitive functions that are mainly affected in these diseases [60]. In addition, although there is still some controversy in the results in humans and in animal models of this disease, it has been shown that neurogenesis is altered and that the deposition of the extracellular Aβ peptide (in the case of AD) is involved in this alteration [63]. On the other hand, PD is also closely related to the process of adult neurogenesis. As in the previous case, in humans, there is still controversy regarding the results [62], but in animal models, it has been observed that, in general, there is a decrease in neurogenesis in brain zones where this process occurs physiologically [64].

### 2.8. Metal Ions Homeostasis Alteration

It has been previously demonstrated an alteration of metal ions homeostasis, including iron (Fe), copper (Cu), and zinc (Zn) in AD and PD. However, at this time, the reasons for this are unclear. In general, it is characteristic an increase and site redistribution of these metal ions levels, together with the appearance of these ions in Aβ plaques and Lewy bodies, the typical protein aggregates of AD and PD, respectively. There are some clues that indicate that this could due to the altered function of regulator homeostasis proteins; it appears that APP, α-syn, neuromelanin, and other proteins not related with these diseases act as indirect or direct regulators of some metal ions homeostasis [65,66,67].

These metal ions homeostasis alteration has cytotoxic consequences on cell survival. On the one hand, the reaction of some metal ions with free radicals or molecular oxygen increases oxidative stress by Haber-Weiss and Fenton reaction. Moreover, some pro-oxidant enzymes (like xanthine oxidase or nitric oxide synthase) can be activated in an indirect way by these metal ions [65]. Additionally, some of these metal ions can interact with the archetypal proteins involved in these diseases and promote their aggregation [66,67]. Lastly, among other cytotoxic consequences, iron’s capacity to activate a programmed cell death known as ferroptosis, which is independent of the oxidative stress and other cell death pathways, is noteworthy [66].

## 3. Novel Treatment Strategies in AD

**Figure 2:**
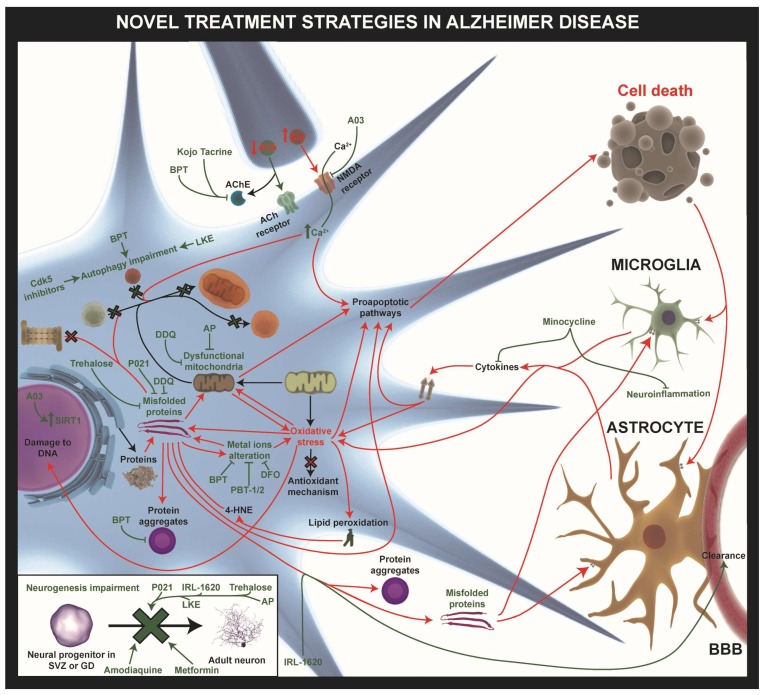
Novel treatment strategies for AD. The most relevant approaches to restore altered pathways in AD are shown with green arrows when processes are improved or T-bars when inhibited. The novel acetylcholinesterase (AChE) inhibitors BPT and Kojo tacrine affect autophagy impairment, misfolded proteins, and their aggregates in the intracellular and extracellular brain environment. A03 compound has an antagonist influence on the NMDAR-mediated pathways involved in increasing *SIRT1* expression. Other target strategies of autophagy impairment are CDK5 inhibitors and small lanthionine ketamine-ethyl ester (LKE) molecules against increased CRMP2 expression. Neurogenesis impairment is improved by P021, IRL-1620, trehalose, metformin, LKE, amodiaquine, and allopregnanolones (AP) (BR297) treatments. IRL-1620 increases the clearance of Aβ in the bloodstream by influencing the Endothelin B (ETB) receptor. P021 and trehalose have positive influences on misfolded proteins. Diethyl(3,4-dihydroxyphenethylamino) (quinolin-4-yl)methylphosphonate (DDQ) and AP (BR297) increase mitochondrial biogenesis and Aβ clearance. Minocycline increases anti-inflammatory responses and decreases pro-inflammatory responses. Metal ions homeostasis alteration can be rescued by PBT-1, PBT-2, 4-(1-benzylpiperidin-4-yl)thiosemicarbazones (BPT) derivatives, and deferoxamine (DFO).

Most of the novel treatments are based on multi-facet strategies against familiar or idiopathic AD. Here, we focus on the recent preclinical targets and novel treatment approaches that are under investigation (Figure 2, Table S1). Nowadays, only five Food and Drug Administration (FDA) approved drugs are on the market and these only diminish the progression of the disease. Four of them, donepezil, galantamine, rivastigmine, tacrine, are based on acetylcholinesterase inhibition, and one of them, memantine, has an antagonist influence on NMDAR [68,69,70]. At present, three clinical trials for AD are still ongoing: gantenerumab (ClinicalTrial.gov identifier: NCT03443973, NCT03444870, NCT02051608, NCT01224106, NCT01760005), crenezumab (NCT02353598, NCT01998841, NCT02670083, NCT03443973, NCT03491150), and aducanumab (NCT01677572, NCT02484547, NCT02477800, NCT03639987) (Table S1). Earlier immunotherapies caused the failure of immunization of these monoclonal antibodies in AD due to unsuccessful clinical efficacy and major safety problems (e.g., amyloid-related imaging abnormalities) when used at high doses. Other underlining factors are variation in their antibodies epitopes as well as a high variability in the recognition of the structural conformation of Aβ species. Accessibility of N-terminus antibodies immunization of Aβ is more prone to success, compared to hydrophobic C-terminus immunization. Also, N-terminus antibodies have more efficient clearing of the aggregates, since bapineuzepam, gantenerumab, and aducanumab provoke microglial activation and phagocytosis. New trials targeting prodromal and early stage of the disease (e.g., gantenerumab, crenezumab, BAN2401, aducanumab) are in the pipeline, since most of previous trails failed. The main reason of this failure is a late intervention in patients when too much Aβ has been accumulated and the Aβ cascade is irreversible [71,72,73]. Therefore, novel treatments are urgently needed, including both single target and multi target drugs therapies that could act on the molecular pathway links to misfolded proteins (Aβ and tau), synaptic integrity, cognitive impairments, autophagy, and mitochondrial dysfunctions (e.g., oxidative stress, peroxidase induced cytotoxicity), as well as pro- and anti-inflammatory responses related to AD. However, non-invasive administration methods for these drug compounds have been taken into account.

### 3.1. Targeting the Excitotoxicity and Misfolding Protein Aggregations

A few newly developed compounds in preclinical research been proven to have similar properties as acetylcholinesterase inhibitor and NMDAR antagonist/agonist that have established use in the clinic. However, they might have beneficial effects on other dysregulated molecular pathways in AD. Here, we focus on the molecular drug targets that have additional activity to that already assumed by the FDA approved drugs.

#### 3.1.1. Novel Acetylcholinesterase Inhibitors for Multi-Target Drug Therapy

In AD, a low level of acetylcholine is an important factor in cognitive impairment. Inhibition of acetylcholinesterase (AChE) has been shown to increase cognitive impairment and most 4-(1-benzylpiperidin-4-yl) thiosemicarbazone (BPT) analogues (except 2,3,4-OH-BBPT) have shown potential to act as moderate AChE inhibitors compared to those that are already clinical available AChE inhibitors, like donepezil. Besides this, various 4-(1-benzylpiperidin-4-yl) thiosemicarbazone (BPT) analogues were tested on the other five major hallmarks related to AD: anti-proliferative activity, metal chelation, oxidative stress, dysfunction of autophagy, and protein aggregation. The pyridoxal 4-(1-benzylpiperidin-4-yl)thiosemicarbazone (PBPT) analogue proved to be the best for all of the five major AD hallmarks. A sixth factor was assessed to determine the feasibility of crossing the blood–brain barrier (BBB) by oral administration following the “Lipinski’s Rule of Five” [74].

The other major factor in AD is the dysregulation of metal ion chelation (copper (Cu^2+^), zinc (Zn^2+^), iron (Fe^3+^)). These ions accumulate within the Aβ plaques, of which Cu^2+^ and Zn^2+^ facilitate the self-aggregation of Aβ_(1–40)_ and Aβ_(1–42)_ peptides [75]. Overall, this study demonstrated (in order of decreasing efficacy) that 2,3-OH-BBPT, 8-OH-QBPT, PCBPT, 2,3,4-OH-BBPT, SBPT, QBPT, and PBPT display the ability to inhibit Cu^2+^-mediated Aβ_(1–40)_ and Aβ_(1–42)_ aggregation. Nevertheless, PBPT analogue and its metal chelators Cu^2+^ and Fe^3+^ have demonstrated low anti-proliferative efficacy, which is a desirable characteristic for a long-term AD treatment. Besides this, PBPT showed a greater ability to inhibit ^59^Fe cellular uptake from ^59^Fe-transferrin complexes by at least 41% compared to the controls. A look at the effect of iron complexes on ascorbate oxidation demonstrated that Fe^3+^ complexes of PBPT, NBPT, 8-OH-QBPT, and 2,3-OH-BBPT analogues inhibited ascorbate oxidation more greatly than control samples. These observations suggest that these ligands have the potential to alleviate the Fe-mediated oxidative stress observed in AD. Also, PBPT and SBPT analogues were able to alleviate hydrogen peroxide-mediated cytotoxicity which shows their potential to prevent oxidative stress in AD [74]

Despite this, the autophagy mechanism, which is dysregulated in AD, plays an important function by eliminating misfolding proteins. However, it was demonstrated that the BPT analogues PBPT, PCBPT, 8-OH-QBPT, and 2,3,4-OH-BBPT increase autophagy flux, while NBPT, 2,3-OH-BBPT, and SBPT inhibit the autophagy degradation pathway. Otherwise, no significant effect of QBPT was observed on the autophagy pathway. This modulation could be a crucial function in increasing the clearance of Aβ aggregated species which may be one of the major problems in AD [74].

Another acetylcholinesterase inhibitor, tacrine, a lost multi-target drug entity, was FDA approved. Unfortunately, it was discontinued after other more prone acetylcholinesterase inhibitors were discovered, due to its severe liver toxicity [70]. New insights have shown that Kojo tacrine (KT2D), a tacrine isoform with similar acetylcholinesterase inhibition was developed. This KT2D was synthetized with antioxidant properties and is less hepatotoxic than tacrine, fully completely selective against AChE, and significantly neuroprotective against Aβ. Further properties of various KT2D racemate mixes need to be tested extensively both in vitro and in vivo to determine their clinical translation properties [76].

#### 3.1.2. Novel NMDA-Receptor Antagonist as an AD Idiopathic Treatment Strategy

The major genetic risk factor in AD is apolipoprotein-E4 (ApoE4), which has a significant potential to reduce *Sirtuin* 1 (*Sirt1*) expression. This sirt1 reduction leads to decreases in the FOXO3-mediated oxidative stress response, Coactivator 1-α (PGC1α)-mediated ROS sequestration, and RARβ-mediated ADAM10 expression and increases in P53-mediated apoptosis, NFκB-mediated Aβ toxicity, as well as the acetylation of tau, which leads to microtubule instability and tau pathology [77]. Various *Sirt1* enhancers have been identified, such as alaproclate, resveratrol, quercetin, fisetin, SRT1720, SRT1460, and A03 racemates [78,79,80,81,82,83,84]. Nevertheless, A03 was described as a non-competitive NMDAR antagonist. A03 has a similar effect on reducing the excitotoxicity as the FDA approved drug memantine. Besides this, memantine does not influence *Sirt1* expression. However, A03 has shown beneficial orally pharmacokinetics profiles in the treatment of *Sirt1* related pathogeneses in AD. In the amyloidogenic pathway, cleavage fragments of sAPPβ as well as toxic Aβ species have been shown to decrease after A03 treatment in transfected cell cultures either expressing ApoE4 or ApoE3. The decrease of sirt1 expression was greater in ApoE4 compared to ApoE3 transfected cultures and was increased by A03 treatment. No similar effect was seen for sAPPα. Unfortunately, in vivo A03 treatment of E4FAD transgenic animals did not show any significant effect on the cleavage fragments Aβ_(1–42)_, sAPPα, and sAPPβ or the sAPPα/sAPPβ ratio in the hippocampal area, which is crucial area for AD pathology. Besides this, long term oral A03 treatment of AD transgenic animals increased *Sirt1* expression in the hippocampus, but not in the frontal cortex, which has been associated with memory improvement. The importance of the above-mentioned compound needs to be further investigated in terms of the relation between sirt1 and sAPPα in dose dependent cases in both preclinical and clinical trials. The *Sirt1* function has a major role related to tau pathology, which is an additional risk for progression during AD. The first-in-class ApoE4 targeted therapeutic, A03, which influences *Sirt1* levels might be a good candidate for preclinical trails in MCI and AD due to its excellent brain bioavailability and promising efficacy after chronic oral treatment [77].

#### 3.1.3. Other Target-Receptor Mediated Treatment Strategies

The endothelin B (ETB) receptors is abundant in the CNS and has been shown to play a role in development and neurogenesis. To influence the receptor-mediated function, a highly specific agonist IRL-1620 was used to target the ETB receptor. Endothelin-1 isopeptide (ET-1) plays a central role in the regulation of cardiovascular functions and regional blood flow [69,85,86] and may be part of the mechanism by which Aβ interferes with vascular function in AD. It has been shown that Aβ upregulates endothelin converting enzymes 1 and 2 (ECE-1 and ECE-2), which results in increased production and release of ET-1 [69,87,88,89]. ECE-1 helps the clearance of Aβ by fragmentation of the peptide [90]. The compound IRL-1620 stimulates the clearance of both ET-1 and Aβ as well as cerebral blood flow which might have a positive influence on the clearing mechanisms of toxic aggregates. Besides this, the compound improves memory deficiency and reduces oxidative stress, which are both caused by Aβ toxicity [69,91,92]. IRL-1620 has been demonstrated to increase neural growth factor (NGF) and synapsin I expression, which are both factors involved in neurogenesis and synaptogenesis and which are altered in MCI and AD pathologies [69,92,93,94].

### 3.2. Targeting Mitochondrial Dysfunction and Related Pathways Involved in AD

Recently, the involvement of mitochondrial dysfunction in AD has been investigated by using the pharmacologically developed compound diethyl(3,4-dihydroxyphenethylamino) (quinolin-4-yl)methylphosphonate (DDQ). DDQ has demonstrated positive effects on mRNA and protein levels related to mitochondrial dysfunction and synaptic dysregulation, which are both related to AD. Besides this, DDQ has an effect on the mitochondrial dynamics, related to fission proteins (DRP1 and Lis1), fusion proteins (Mfn1 and 2), and Aβ interactions. DDQ has shown a better docking score than other single existing molecules, like MitoQ, Mdivi1, and SS31. One advantage of DDQ as a novel target is that it binds to the active sites of Aβ and DRP1, inhibiting the Aβ and DRP1 complex formations. The mRNA and protein levels of mitochondrial (*PGC1α, Nrf1, Nrf2, TFAM, DRP1, Fis1, Mfn1 and 2*) and synaptic activity (Synaptophysin, PSD95, synapsin1 and 2, synaptobrevin1 and 2, synaptopodin, and GAP43) were investigated after Aβ-induced pre-treatment or post-treatment with DDQ. The mRNA and protein levels (*PGC1α, Nrf1, Nrf2, and TFAM*) were significantly increased after Aβ incubation followed by DDQ treatment. Besides this, reductions of mitochondrial fission proteins (DRP1 and Fis1) and increases of mitochondrial fusion proteins (Mfn1 and 2) were observed after DDQ treatment. Despite this, pre-treatment with DDQ followed by Aβ treatment increased mitochondrial biogenesis mRNA (*PGC1α, Nrf1, Nrf2, and TFAM*) levels, which suggests that DDQ pre-treatment could serve as prevention agent in AD. DDQ pre-or post-treatment induced Aβ incubation led to a downregulation in mitochondrial fission protein activity (DRP1 and Fis1) and upregulation of fusion activity (Mfn1 and Mfn2). This led to the conclusion that DDQ pre-treatment reduces fission activity (DRP1 and Fis1) and enhances fusion activity (Mfn1 and 2) in the presence of Aβ. However, DDQ treatment-induced Aβ incubation also has a potential enhancing effect on synaptic activity which is downregulated by Aβ pathology. The reduction of DRP1 and Aβ complexes is stronger in DDQ pre-treated compared to post-treated cells. The reduction between Aβ and DRP1 interaction due to DDQ treatment leads to a reduction in mitochondrial fragmentation and maintains the normal count, normal length and normal function of mitochondria, and it might be protective against the Aβ plaque load. Nevertheless, DDQ treatment has been demonstrated to play a neuroprotective role in Aβ toxicity by significantly reducing Aβ_(1–42)_ levels and increasing Aβ_(1–40)_ levels. Despite this, DDQ enhances mitochondrial function and increases cell viability, which leads to an increase in mitochondrial ATP and cytochrome oxidase activity, as well as a reduction in free radicals and oxidative stress, which is dysregulated in AD [95].

### 3.3. Targeting Autophagy

Autophagy dysregulation has been linked indirectly to AD using microtubule-associated protein CRMP2 (collapsin response mediator protein-2) modulation. CRMP2 seems to have same features as tau protein, but, besides this, CRMP2 undergoes profound posttranslational modifications in the brain. CRMP2 is an important adaptor protein that is involved in vesicle trafficking, amyloidogenesis, and autophagy. Tau protein did not show similar involvement in these molecular pathways [96]. Actually, CRMP2 was discovered to be a mediator of neurite retraction and neuron polarization during semaphorin signaling [97]. Besides this, tau has a direct effect and CRPM2 has an indirect effect on the stabilization of actin-based microfilament networks. The difference between CRMP2 and tau is that CRMP2 is involved in endosomal-lysosomal trafficking and autophagy [98]. Although CRMP2 has a highly binding affinity to endocytic adaptor protein (Numb) and MICAL-like protein 1 (MICAL-L1), which are involved in intracellular vesicle movement, as well as the amyloidogenic processing of APP which occurs by endosomal trafficking, it has been speculated that CRMP2 becomes functionally depleted in AD pathology due to both combination of the hyper-phosphorylation of tau; sequestration into nascent neurofibrillary tangles; and oxidative post-translational modification of CRMP2. This includes different multi-facet CRMP2 dependent processes, including amyloidogenic APP trafficking through early and recycled endosomal compartments. Also, it has been demonstrated that the knock-down of CRMP2 expression influences the autophagy flux [99]. Nevertheless, the CRMP2-binding small molecules lanthionine ketamine-ethyl ester (LKE) normalize CRMP2 phosphorylation, reducing the Aβ burden and phosphorylating tau in AD in vitro and in vivo models. Even some derivations of LKE can functionally enhance CRMP2 to promote growth factor-dependent neurite outgrowth as well autophagy-related processes [96]. Despite this, long term LKE treatment increases beclin-1 protein, which is another autophagy-related protein that is downregulated in MCI and AD, which negatively influences the Aβ flux. Besides this, Glia-derived neurotrophic factor (GDNF) can increase CRMP2 expression, which results in microtubule stabilization and enhanced neurite outgrowth [99,100].

The activity of mTORC1, the gate-keeper of autophagy, can increase CRMP2 expression which shows that CRMP2 might be a good therapeutic target [101,102]. Nevertheless, the CRMP2 phosphorylation signaling pathway could be modified by a pharmacological inhibitor of Cdk5, which is under development and which can target the glycogen synthase kinase-3 (GSK3), which is the major compound for regulating the hyper-phosphorylation of either tau or CRMP2 [103,104]. An upstream therapeutic target that might influence CRMP2 expression, as can be seen by axon repulsion factor semaphoring 3A (Sema3A), which binds to the neuropilin-1 receptor (NRP1) and plexin-A co-receptors; its relation in AD needs to be further exploited [105,106]. Besides that, tau was discovered earlier than CRPM2, and tau is more prone to form non-dissociable high molecular weight complexes than CRMP2. Also, CRMP2 and phosphorylated-CRPM2 (pCRMP2) are not prone to aggregate or form filaments like tau, which later on accumulate to form or associate with NFT-related proteins. Despite this, tau mutations are neuropathic in humans, whereas a CRMP2 mutation has not been discovered yet [96]. Nevertheless, CRMP2 hyper-phosphorylation is a very early event that occurs prior to Aβ accumulation or tau hyper-phosphorylation [107]. This effect of CRMP2 hyper-phosphorylation is a downstream consequence of altered non-amyloidogenic APP processing and perhaps been a more prominent function than tau dysregulation in disorders involving both amyloidopathy and NFT aggregation [108]. 

### 3.4. Targeting Neuroinflammation

Neuroinflammation is seen as an early event in AD, and drug agents involved in targeting this process could slow down disease progression. Recently, Minocycline, a tetracycline antibiotic, which interferes with the symptoms of neuropsychiatric disorder related to AD. Minocycline has been shown to decrease proinflammatory cytokines, like tumor necrosis factor α (TNF-α) and interleukin 1β (IL-1β), and increase anti-inflammatory cytokines, like interleukin 10 (IL-10), in Aβ_(1–42)_-treated animals compared to control rats [109]. This neuropsychiatric compound can be considered a new treatment possibility to act against the early effects of AD neuropathology. 

### 3.5. Activation of Neurogenesis and Neuronal Survival Pathways

Neurogenesis pathways are downregulated in AD, and various attempts have been made to improve the neurogenic survival pathways in neurodegeneration by interfering with Aβ and tau related mechanisms, boosting dendritogenesis or synaptogenesis and relevant metabolic processes involved in AD pathology [86,110]. Beneath, some novel compounds that are under preclinical research as possible new treatment strategies are discussed. Nowadays, interest is growing in developing more orally bioavailable compounds as a non-invasive therapeutic tool. These compounds are easily degraded and have the ability to cross the BBB.

#### 3.5.1. Orally Bioavailable Compounds

The first orally bioavailable neurotrophic factor is a tetra peptide (P021), which is derived from ciliary neurotrophic factor (CNTF), and which has a suitable biodegradation level and is able to cross the BBB easily. The general functions of P021 are to inhibit leukemia inhibitory factor (LIF) signaling and increase brain-derived neurotrophic factor (BDNF) expression. However, P021 treatment has been able to rescue dendritic and synaptic deficits by boosting neurogenesis, preventing neurodegeneration, Aβ, and tau pathologies, rescuing cognitive impairment in AD transgenic mice, as well as markedly reducing age-related mortality in rat. Besides this, P021 inhibits the GSK3β pathway through the phosphorylation of ser-9 by BDNF. This inhibition has direct effects on Aβ and Tau pathology. Both P021 as well as its parent molecule peptide 6 can increase neurogenesis and synaptic plasticity, which improve cognitive performance in AD transgenic animal models, even in the late stage of the disease progression. Nevertheless, no major side effects have been reported. P021 treatment could serve as an additional therapy strategy to act against AD pathology [110].

The second orally bioavailable compound which can be classified as a natural disaccharide, also called trehalose, seems to have a protective role in denaturation and conformational protein changes in neurological disorders [111]. Unfortunately, there is a lot of controversy about trehalose and its derivatives in neurological pathologies. Nevertheless, the major factor in AD is the dysregulation of Aβ protein. It has been reported that Aβ aggregation is inhibited by trehalose and other derivatives [112]. The other major factor involved in AD that was described earlier is metal ion chelation dysregulation. It has been reported that trehalose and its derivatives seem to have a positive influence on Aβ aggregation [75]. Due to this previous relevance, trehalose was studied in the transgenic Tg2576 mouse model for AD [112]. It was shown that intracerebral injection of trehalose improves cognitive impairment in the APP/PSEN-1 model [111]. Unfortunately, this was based on the invasive administration route. However, no statically significant improvement of cognition has been shown by the non-invasive oral administration of trehalose and its derivatives. Also, trehalose treatment did not show any significance in reducing metal ion induced Aβ aggregation, amyloidogenic APP levels, and Aβ fragments, as well as showing no influence on autophagy flux in both the cortex and hippocampus. Nevertheless, trehalose treatment seems to significantly increase the neuromigrating protein doublecortin (DCX), a surrogate indicator for neurogenesis, as well as increasing synaptic activity, especially presynaptic vesicle marker synaptophysin, in the hippocampus and in the cortex [112]. Trehalose treatment seems to increase progranulin expression in both the hippocampus and the cortex. In AD, progranulin, a regulator of neuronal growth and survival, has shown a protective effect against Aβ neurotoxicity [112,113,114].

#### 3.5.2. Other Pro-Neurogenic Compounds Related to Metabolic Disorder Linked to AD

Metabolic disorders like type II diabetes could be genetically linked to AD. In both of these disorders, alterations in neurogenesis are seen. Besides this, the roles of insulin and insulin growth factor, which are important factors in diabetes, have been demonstrated to influence neurogenesis in ex vivo and hippocampal cultured cell lines and might play a crucial role in AD pathology [115]. Compared to an FDA approved drug (donepezil), metformin, a type II anti-diabetic drug, has demonstrated both pro-neurogenic potential as well as higher spatial improvement in cognitive impairment in an aluminium chloride (AlCl_3_)-induced (AlCl_3_·6H_2_O) mouse model, a metabolic model for neurodegeneration [68,116]. Metformin-treated aluminium chloride (AlCl_3_)·6H_2_O-induced animals seems to have an irreversible increase in neurogenesis factors (DCX and Neuronal Nuclei (NeuN)), and this can be explained by the effect of metformin on the insulin-mediated Akt signaling pathway compared to donepezil-treated animals. Besides this, metformin has been shown to normalize the aberrant hippocampal proteome signature. Increased levels of the synaptosomal proteins such as calcium/calmodulin-dependent protein kinase type II subunit α (KCC2A), Synapsin-1 (SYN-1), and gamma soluble NSF attachment protein (SNAG) were observed in this metabolically-induced model treated by metformin. All of them play roles in molecular mechanisms related to learning and memory in AD. Moreover, the upregulation of SYN-1 and KCC2A in this metformin-treated metabolically-induced model indicates that metformin facilitates memory formation through synapse plasticity and a possible BDNF-mediated increase of neurogenesis. Other proteins, like ubiquitin-like modifier activating enzyme 1 (UBA1), that are involved in ubiquitin proteasome system (UPS) dysfunction in AD, were shown to increase after metformin treatment. This can be interpreted as a protective response to abnormal or aggregated proteins. Other metabolic proteins, glutathione S transferase 1 (GSTM1) and aconitrate hydratase (ACON) were downregulated in AlCl_3_·6H_2_O groups, which is directly linked to an increase in oxidative stress. Following metformin treatment, this downregulation was restored. Alterations in the metabolism-associated proteins in the AlCl_3_·6H_2_O-treated group and their restoration by metformin strengthens the idea that AD could be a metabolic disorder. Metabolic drugs could be an alternative therapeutic multi-target strategy to improve the pro-neurogenic effect in AD. Besides this, SYN-1 upregulation-mediated BDNF activation may represent one of the underlying mechanism(s) of metformin-mediated neurogenesis and needs to be further investigated [116].

### 3.6. Targeting Metal Ions Homeostasis Alteration

Different types of strategies have been developed to recover the metal ions homeostasis alteration. One of these strategies is to decrease the peripheral levels of these metal ions to indirectly decrease their levels in the brain. However, there is not really conclusive evidence of this theory and it could be dangerous to employ this strategy to the entire organism due to the possibility of causing peripheral metal ions deficiency [66]. This is the reason why the drug development against metal ions homeostasis alteration is focused on developing metal-protein-attenuating compounds (MPAC). The MPAC strategy promotes a better distribution of metal ions by the interference of abnormal interaction between some proteins and metal ions in these diseases, allowing for better clearance by the normal endogenous clearance cell processes [65]. Nevertheless, one of the main problems of MPAC development is the difficulty of these drugs to cross the BBB due to their high molecular weight [66].

One example of MPAC drugs is 5-Chloro-7-iodo-quinolin-8-ol (clioquinol or PBT-1), a chelator of Cu, Zn, and Fe which is able to cross the BBB. Several studies have been performed analyzing the effect of this drug in AD, including a phase II clinical trial, in which it was confirmed as effective as a disease-modifying therapy as clioquinol. This drug specifically prevents the cognitive decline and decreases the levels of Aβ42 in plasma and amyloid deposition in the brain [65,66]. Other developing MPAC treatments against AD are PBT-2 (chelator of Cu, Zn, and Fe) or deferoxamine (DFO), an Fe chelator, which also improves the cognitive decline and reduction of amyloid deposition [117]. 

### 3.7. Other Targets

Previous data showed that Aβ and abnormal tau protein alter neurosteroidogenesis in AD models [118,119]. Natural neurosteroids allopregnanolones (AP) are synthesized following a transformation that involves 5α-reductase activity, which converts progesterone into 5a-dihydroprogesterone (5α-DHP), and also 3α-hydroxysteroid oxidoreductase, which transforms 5α-DHP into AP but also reversibly converts AP back to 5α-DHP. It has been demonstrated to improve steroidogenesis [120,121]. Despite this, various selected AP derivatives, especially BR297, appear to be the best to mimic the effect of AP on bioenergetics in cellular models. AP and BR297 seem to protect against H_2_O_2_-induced cell death by improving mitochondrial bioenergetics by ameliorating cellular ATP production and reducing ROS generation under oxidative stress. Depending on the structural chemical modification of these AP derivatives, they can either increase mitochondrial respiration or induce neurogenic and neuroprotective effects by preventing neuronal cell death without causing the stimulation of cell proliferation [122]. Thus, it is really important to know what the effects of AP derivatives are on the receptor-mediated effects of bioenergetics and cell survival. Here, we focus on the concepts related to AD. The well-known gamma-aminobutyric acid-A receptor (GABA-A) might be modulated through AP derivatives, but the involvement of only GABA-A receptors can be hardly justified. For example, AP promotes the cell proliferation of neural progenitors, induces hippocampal neurogenesis, decreases cerebral Aβ production, and reverses memory deficits in AD triple transgenic mice through acting on the L-type Ca^2+^ channels and GABA-A receptors [123,124,125,126].

## 4. Novel Treatment Strategies in PD

### 4.1. Targeting α-Synuclein Aggregation

**Figure 3:**
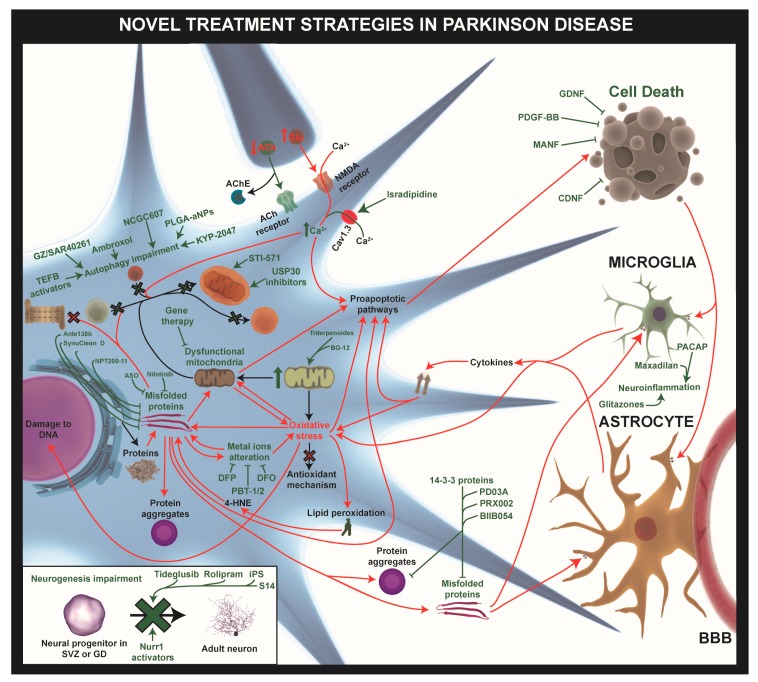
Novel treatment strategies in PD. The most relevant approaches to restore altered pathways in PD are shown with green arrows when processes are improved or T-bars when inhibited. The compounds used to target α-Syn aggregation are antisense oligonucleotides against these protein aggregates (ASO), oligomer modulators (Anle138, SynucleanD and NPT200-11), and cAbl inhibitors (Nilotinib). Pathogenic α-Syn spreading can be avoided by 14-3-3 protein activation and the use of active (PD03A) or passive (PRX002 and BIIB054) immunization. Autophagy impairment is targeted by stimulation with TFEB activators or Beclin-1 activity inducers (KYP-2047), but also by the activation of lysosomal activity with GBA activators (Ambroxol), glucosylceramide synthase (GCS) inhibitors (GZ/SAR40261), GCase activators (NCGC607), and acid nanoparticles (PLGA-aNPs). Ca^2+^ channel inhibitors (Israpidine) preserve physiological levels that are altered in PD. Mitochondrial biogenesis can be activated with Nrf2 activators (BG12 and triterpenoids). Nip3-like protein X (NIX) activators, cAbl inhibitors (STI-571), and deubiquitinases (DUBs) inhibitors (USP30 inhibitors) increase degradation of damaged mitochondria. PD-linked mutations affecting mitochondria can be modified by gene therapy. Using a pituitary adenylate cyclase-activating polypeptide (PACAP) receptor agonist (Maxadilan), phosphodiesterase 7 (PDE7) inhibitors (S14), or peroxisome proliferator-activated receptor-γ (PPARγ) inhibitors (glitazones) could target neuroinflammation. The activation of neuronal survival pathways can be improved by administration of neurotrophic factors (GDNF, MANF, CDNF, and PDGF-BB). Nuclear receptor related 1 (Nurr1) activators, cAMP response element binding protein (CREB) activators (Rolipram), glycogen synthase kinase-3 (GSK3) activators (Tideglusib), or induced pluripotent stem (iPS) cell transplantation might restore neurogenesis impairment.

As mentioned above, α-Synuclein aggregation is one of the hallmarks of PD, and there is a number of processes that can be the target for therapeutic strategies to avoid these detrimental effects of α-Syn: (1) reducing α-Syn synthesis, (2) avoiding protein accumulation (by increasing its degradation), (3) lowering protein misfolding and aggregation, and (4) blocking cell-to-cell transmission (Figure 3, Table S2).

Related to the first aim, the principal approach to reduce α-Syn synthesis is the use of antisense oligonucleotides to target *α-Syn* mRNA. For example, a decrease in hippocampal and cortical α-Syn has been observed in mice when direct siRNA infusion was performed [127]. Furthermore, in a mouse model overexpressing α-Syn, the injection of α-Syn containing exosomes reduced this protein aggregation in the Substantia Nigra pars compacta (SNpc) [128]. With these results, ASO-mediated therapies, which are now in clinical trials for other neurodegenerative diseases, may be effective to target αSyn synthesis [129].

With respect to the second aim, some new approaches are being developed to increase α-Syn degradation. Among them is the activation of the autophagy pathway, which allows α-Syn clearance (this topic will be discussed later). Another option to increase the degradation of α-Syn is modulating the lysosomal membrane protein glucocerebrosidase (GBA) pathway. Mutations on GBA are a risk factor for developing PD, and a decrease in this protein’s activity is thought to lead accumulation of α-Syn. For this reason, phase 2 clinical trials with a GBA activity enhancer drug, Ambroxol, are currently in process to evaluate the safety, tolerability, and pharmacodynamics of this drug (ClinicalTrial.gov identifier: NCT02941822). However, an alternative to modulating the GBA pathway is the inhibition of glucosylceramide synthase (GCS), given that glucosylceramide is a substrate of GBA and is hypothesized to stabilize α-Syn oligomers [130]. Studies with GZ/SAR40261, an inhibitor of GCS, are now in phase 2 clinical trials for the treatment of PD patients carrying a GBA gene mutation (NCT02906020).

Regarding the third aim, there are a number of studies that have tried to reduce α-Syn misfolding and aggregation. One of them aimed at reducing the C-terminal truncation present in α-Syn which makes it prone to aggregation [131]. This C-terminal truncation is due to inflammatory protease caspase-1, which has been described as being involved in the aggregation mechanism of α-Syn [132]. For this reason, inhibiting Caspase-1 could be a good approach to decrease α-Syn cleavage and toxicity. This was studied in a proteolipid protein α-Syn (PLP-SYN) mouse model of multiple system atrophy, a disorder associated with α-Syn accumulation in oligodendrocytes, showing an improvement in α-Syn pathology and also in motor deficits [133]. Another strategy is the use of oligomer modulators such as 3-(1,3-benzodioxol-5-yl)-5-(3-bromophenyl)-1H-pyrazole (anle138b), which directly binds to a structure-dependent epitope of α-Syn and strongly inhibits protein oligomerization in vitro and in vivo. This compound has good oral bioavailability and is able to cross the blood–brain barrier [134], so it is a good candidate for clinical trials.

Furthermore, Pujols et al. recently described one of the newest small molecules able to reduce α-Syn aggregation, called SynuClean-D [135]. This compound significantly reduces aggregation of α-Syn in vitro in neuroglioma cells and in *Caenorhabditis elegans* PD models. The most promising characteristic of this molecule is its ability not only to decrease α-Syn aggregation but also to rescue DA neurons from α-Syn-induced death. Also, some compounds that avoid α-Syn aggregation are in clinical trials. This is the case for the novel small molecule NPT200-11 which targets pathogenic α-Syn, stabilizes its conformation, and prevents its oligomerization. This compound is now in phase 2 clinical trials to assess drug dynamics, efficacy, and safety (NCT02906020). Other mechanism involving the tyrosine kinase c-Abl to reduce α-Syn aggregation has been suggested based on preclinical evidence, since the inhibition of c-Abl reduces α-Syn aggregation and therefore neuropathology in vivo [136]. According to these data, a specific inhibitor of c-Abl kinase activity called Nilotinib is now in phase 2 clinical trials (NCT03205488).

Finally, concerning the fourth aim, approaches to reduce α-Syn spreading are related to passive (use of antibodies against protein) or active (vaccination-based using full-length protein or short peptides) immunization. For instance, antibodies against C-terminal truncated α-Syn or wild type protein in vitro and in mouse models of α-Synucleopathy decrease its propagation and avoid α-Syn toxicity effects [137,138]. However, new approaches to decrease α-Syn cell-to-cell transmission and also the level of this protein in cells consist of the modulation of the action of glial cells. One example is the enhancement of astrocytic capture and degradation of α-Syn fibrils [139]. The recent findings that 14-3-3 proteins (with chaperone and trafficking regulation functions) are able to reduce cell-to-cell transfer of α-Syn and decrease protein-derived toxicity in cell and animal models of α-Syn aggregation are also encouraging [140]. It is also important to mention that some clinical trials currently in phase 2 involve the targeting of α-Syn by active immunization with the vaccines AFFITOPE^®^PD01A and PD03A, and passive immunization with antibodies against α-Syn PRX002 (NCT02157714) and BIIB054 (NCT03318523). 

### 4.2. Targeting Mitochondrial Dysfunction and Oxidative Stress

As mentioned above, mitochondrial activity is altered in the context of PD, and this is mainly due to α-Syn aggregation, which can affect the structure and function of this organelle, as well as PD-linked mutations in PINK1, parkin and DJ1 proteins, which alter mitochondrial quality control and mitophagy processes, and increase sensitivity to oxidative stress.

Knowing that, there are a number of possible ways in which mitochondrial alteration can be targeted for the treatment of PD. In this review, we present some of them: (1) mitophagy activation to eliminate damaged mitochondria, (2) an increase in mitochondrial biogenesis, and (3) gene therapies targeting PD-linked gene mutations (Figure 3, Table S2). 

Regarding the first aim, mitophagy increase can be achieved by the activation of parkin. The above-mentioned tyrosine kinase c-Abl phosphorylates parkin, inhibiting its enzymatic activity. It has been described in cell and animal models of PD based on the use of the toxin 1-methyl-4-phenyl-1,2,3,6-tetrahydropyridine (MPTP) that STI-571, a c-Abl inhibitor, prevents the phosphorylation of parkin, maintaining it in a catalytically active and protective state (Ko, 20823226). Moreover, a novel finding is that both genetic and pharmacological induction of the mitochondrial autophagy receptor Nip3-like protein X (NIX) restore mitophagy in PD patient-derived fibroblasts [141]. Another way to activate mitophagy is by the inhibition of deubiquitinases (DUBs), given that a large number of them regulate mitochondrial function [142,143,144,145]. One example is USP30, the only DUB that is exclusively localized to mitochondria [146], which deubiquitinates parkin substrates. USP30 inhibition has been seen to enhance parkin-mediated mitophagy [147].

Moving on to the second aim, an increase in mitochondrial biogenesis can be also an interesting target for restoring the mitochondrial pool in damaged cells. It has been recently shown that BG-12 (dimethyl fumarate) increases mitochondrial biogenesis in mice and multiple sclerosis patients by activation of the transcription factor Nuclear factor (erythroid-derived 2)-like 2 (Nrf2) [148]. Other activators of the Nrf2 pathway called triterpenoids have been associated with protection of DA neurons in mouse PD models based on the use of MPTP toxin [149]. Another possible target is peroxisome proliferator-activated receptor-γ (PPARγ) coactivator 1-α (PGC1α) because of its role in inducing mitochondrial biogenesis [150]. For example, quercetin showed protective effects in rodent models of neurodegeneration by increasing mitochondrial function [151], which can be another option for new drug development for PD treatment.

The third approach that may be carried out to improve mitochondrial activity in PD is gene therapy. In a variety of preclinical studies in PD animal models, it has been described that gene therapy consisting of the overexpression of PINK1 and parkin proteins has neuroprotective effects by restoring or enhancing mitochondrial function and bioenergetics. However, translation of these findings from animal models to the clinic is challenging due to the inability of animal models to collect all phenotypic characteristics of PD and the limitations with respect to the determination of the initial dose, direct target success quantification, and assessment of transgene production and localization [152]. For this reason, the next steps for gene therapy application against PD will require efforts to translate findings from animal models of PD to clinical benefits.

Finally, it is also important to mention that oxidative stress is linked to mitochondrial function, not only because mitochondria produces ROS, but also because these reactive species can deteriorate mitochondrial components and functions. For this reason, targeting oxidative stress by the use of antioxidant drugs may also confer protection to mitochondria. This improvement in mitochondrial activity has been observed in several preclinical studies using mitochondria-targeted antioxidants, such as vitamin E, coenzyme Q10 (coQ10), or urate [153]. However, recent clinical trials for creatine and coenzyme Q10 have not demonstrated disease-modifying benefits in patients with PD [154,155]. Another example is the positive effect of the antioxidant MitoQ, a redox active ubiquinone targeted to mitochondria, which was addressed in animal models of PD [156] and AD [157]. However, clinical trials of the treatment with MitoQ in PD patients showed that this compound does not slow the progression of the disease [158]. Altogether, these results indicate that oxidative stress is a downstream effect of mitochondrial dysfunction rather than a direct cause of PD-related neurodegeneration. 

### 4.3. Targeting Autophagy

Autophagy pathway alteration is an important pathogenic characteristic of PD, and it is linked to α-Syn aggregation as this is one of the fundamental mechanisms by which this protein is cleared. Thus, targeting autophagy by chemical or genetic means could be beneficial to neuronal survival (Figure 3). However, to successfully apply this strategy, it is important to know which phase of the autophagy process is altered in the disease. The reason for this is that deficits in the initiation step may be compensated by autophagy inducers, but if late steps (autophagosome clearance) are affected, these inducers may have detrimental effects, causing overload of the cellular degradation system [159].

One promising way to restore autophagic flux is by modulating the expression or activity of transcription factor EB (TFEB) which regulates the expression of genes involved in autophagy and lysosome synthesis [160], and its activity is altered in the context of α-Syn pathology [161]. Related to this, some studies have shown that genetic or pharmacological stimulation of TFEB is neuroprotective in in vitro and in vivo models of PD [162,163]. However, this is not the only reason for targeting TFEB in PD, given that this transcription factor is also involved in mitochondrial quality control mechanisms, driving the elimination of dysfunctional mitochondria [164].

Autophagy stimulation can be also achieved by the activation of upstream signaling pathways, for example, by the use of AMP activated protein kinase (AMPK) activators, such as metformin and resveratrol, which leads to the inhibition of mammalian target of rapamycin complex (mTORC1) and the initiation of autophagy. However, this approach is problematic, given that these upstream signaling proteins are involved in many other pathways, and because the selectivity of AMPK activation agents for autophagy is limited [165]. For this reason, one solution is to target components of the autophagic pathway in a selective way. This is the case for Beclin-1, a protein that, under activation, leads to autophagosome formation in an mTORC independent way. It has been described that Beclin-1 overexpression was able to reduce the accumulation of α-Syn in cell and mice models overexpressing α-Syn [166,167]. However, not many studies have tested pharmacological Beclin-1 stimulation in PD models [165]. Thus, the proposal that inhibition of prolyloligopeptidase (PREP) induces autophagy in a Beclin-1 dependent way, is a more current aim. The use of KYP-2047 inhibitor of PREP was shown to reduce α-Syn aggregates in cell models and α-Syn transgenic mice [168]. Another way of selectively activate autophagy is by increasing lysosomal activity. To get this, novel strategies have been studied consisting of the use of acid nanoparticles of poly-DL-lactide-co-glycolide (PLGA-aNPs), which are able to stimulate lysosomal degradation by lowering its pH [169]. Some studies using this technique have shown a reversal of lyosomal dysfunction in cellular PD models and a decrease in DA cell death in MPTP treated mice [169]. Another approach is the direct targeting of specific lysosomal enzymes, such as glucocerebrosydase (GCase), to stimulate lysosomal content degradation [165]. To achieve this, some small-molecule chaperones are being developed to correct the folding of GCase, thereby enhancing its activity [170]. One of the most recent ones is NCGC607, a small-molecule non-inhibitory chaperone of GCase that has been shown to recover its activity and to reduce α-Syn levels in iPSC-derived DA neurons [171].

Finally, it is important to mention that targeting autophagy can be dangerous given that this process can have beneficial or detrimental effects depending on the specific context [172]. 

### 4.4. Targeting Intracellular Ca^2+^ Homeostasis Alteration

One important characteristic of dopaminergic neurons in the *SNpc* is that they have a Ca^2+^-dependent pace-maker, which is an autonomous mode of discharge that increases Ca^2+^ levels in the cytosol [173]. This mechanism is necessary to maintain a basal dopaminergic tone in the striatum; however, it may confer a specific vulnerability of DA neurons to excitotoxic stress, because these neurons have a low Ca^2+^ buffering capacity [17]. Thus, DA neuron survival depends on the maintenance of Ca^2+^ in appropriate levels [173,174]. However, cytosolic Ca^2+^ overload may occur in these cells in the context of PD due to sustained activation of NMDAR as a consequence of the increased activity of subthalamic nucleus glutamatergic inputs and also to other altered mechanisms in the cell [17]. This can lead to oxidative stress, possibly due to mitochondrial Ca^2+^ overload and dysfunction [175]. For this reason, alleviating this increase in Ca^2+^ load in DA cells by the use of L-type Cav channel blockers may be helpful in PD (Figure 3). For instance, isradipidine, which almost specifically blocks Cav1.3 channels, is able to reduce basal and toxin-induced mitochondrial oxidative stress [173,175] and is currently in phase 3 clinical trials to determine whether it is effective in slowing the progression of PD (NCT02168842).

### 4.5. Targeting Neuroinflammation

Neuroinflammation is characterized by the activation of glial cells that release pro-inflammatory and toxic factors (Figure 1). A large number of studies in previous decades have described the link between neuroinflammation and PD pathology [176,177,178]. For this reason, targeting microglia activation and the pro-inflammatory cytokines production using drugs or neuroprotective substances could be another interesting pathway to consider for PD treatment. In the last 15 years, a large number of studies have identified several different immunomodulatory agents that protect DA neurons from degeneration and death in animal models of PD. All of them were shown to be effective in reducing the motor deficit and alleviating DA neurotoxicity and also in preventing the decrease of dopamine in those models [179].

However, in this paper, we focus our attention on some novel therapeutic approaches targeting neuroinflammation (Figure 3). One of them is the use of pituitary adenylate cyclase-activating polypeptide (PACAP) that activates pathways regulated by cAMP, also linking this compound to neurotrophic factor signaling in neurons [180]. This peptide has strong anti-inflammatory action and can counteract the effects of pro-inflammatory cytokines produced by microglial cells [181,182]. PACAP is able to cross the blood–brain barrier but has a short half-life in tissue. Due to this, PACAP-receptor agonists, such as maxadilan, or peptide fragments could be more useful [183,184]. However, it is important to state that PACAP has many targets, as it affects various signaling pathways, so its administration could produce side effects. Another possible target to avoid neuroinflammation and stimulate neuroprotection is phosphodiesterase 7 (PDE7). Our group has shown that different inhibitors of PDE7 are potent neuroprotective and anti-inflammatory agents in some animal models of neurodegenerative disorders, including PD [185,186,187]. Moreover, we have demonstrated that PDE7 silencing in *SNpc* using specific shRNAs against this enzyme significantly protects DA neurons and improves motor function in lipopolysaccharide and 6-hydroxydopamine (6-OHDA) lesioned mice [188]. The last target mentioned here that decreases neuroinflammation is (PPARγ). Drugs such as the thiazolidone diones (glitazones), rosiglitazone, and pioglitazone, which are commonly used in the treatment of type 2 diabetes, may also have potential for the treatment of PD [189]. According to this, some studies have demonstrated the beneficial effects of these drugs coupled with reduced neuroinflammation and lower levels of microglial-produced pro-inflammatory cytokines [190,191]. Interestingly, a recent epidemiological study showed that people with type 2 diabetes had lower incidence of PD when they were treated with glitazone drugs [192]. However, clinical trials in PD patients failed to show any reduction in disease progress following the use of pioglitazone [193]. Related to this, a recent study in a 6-OHDA rat model of PD showed that pioglitazone also has a neuroprotective effect by enabling hippocampal neurogenesis [194]. These findings demonstrate that glitazones are able to affect a variety of cell processes. 

### 4.6. Activation of Neurogenesis and Neuronal Survival Pathways

In PD, dopaminergic cell death is the result of the alterations in the different previously described and is the cause of the symptoms observed in patients with this disorder. Promoting DA neuronal survival by the use of neurotrophic factors or by activating the neurogenesis process are interesting approaches for the restoration and survival of this neuronal population (Figure 3).

First, we are going to give a general view of some neurotrophic factors that are considered possible targets for modifying PD progression. Neurotrophic factors are a family of secreted proteins that play key roles in neuronal survival and neuroplasticity. It has been described that these proteins can be upregulated together with their receptors in pathogenic conditions in the brain which corresponds with the notion that these factors are protective and enhance brain plasticity, thus avoiding brain damage [195,196]. According to this, neurotrophin delivery or treatments that increase neurotrophic factor levels in the brain are able to correct oxidative damage and mitochondrial dysfunction, induce anti-apoptotic factors, stimulate cell genesis, synaptogenesis, and axonal sprouting, and even reduce microglial activation [196,197,198]. Among these factors, one of the most studied is the glial cell line-derived neurotrophic factor (GDNF) which has been shown to have neuroprotective and neurorestorative effects in PD animal models; however, it is challenging to achieve such neuroprotection in clinical trials on PD patients [199]. Two newer neurotrophic factors that could be targets for the treatment of PD are the mesencephalic astrocyte-derived neurotrophic factor (MANF) and the cerebral dopamine neurotrophic factor (CDNF), which are localized in the ER, and their expression and secretion is regulated by ER stress [200]. It has been described that CDNF and MANF are able to efficiently protect and repair dopamine neurons in a rodent 6-OHDA PD model [201,202]. Given these results, a phase 1 clinical trial is currently underway to test the safety of brain infusion of CDNF in PD patients (NCT03775538). Another recent target to increase neuronal survival is the platelet-derived growth factor (PDGF) that is present in many tissues with different isoforms [203]. The PDGF-BB isoform was shown to be protective in cultured DA neurons [204] and to induce functional recovery in 6-OHDA rodent models, not by protecting DA neurons, but by stimulating neurogenesis in the subventricular zone [205]. These results inspired a pilot clinical study consisting of intracerebroventricular PDGF-BB delivery in PD patients, which was shown to have good tolerability and resulted in higher levels of dopamine transporter binding in the putamen [206].

Secondly, we want to briefly introduce another strategy for the treatment of PD, which is DA neuron restoration in damaged regions by neurogenesis stimulation or by the use of induced pluripotent stem cells (iPS cells). One way of stimulating DA neurogenesis in the adult brain is through the activation of the transcription factors involved in this process. This is the case for nuclear receptor related 1 (Nurr1), a key regulator of midbrain DA neurons [207]. Nurr1 agonists showed neuroprotective effects on midbrain DA neurons and an improvement in behavioural deficits in a 6-OHDA rat model of PD [207]. Furthermore, some PD-related pathological features can alter neurogenesis, such as the increase in α-Syn levels, which is associated with a negative impact on adult neurogenesis and dendritic development in newborn neurons. Activation of the cAMP response element binding protein (CREB) pathway by rolipram, a phosphodiesterase inhibitor, led to partial improvement of the dendrite outgrowth defect in mice overexpressing α-Syn [208]. Furthermore, it has been recently published that PDE7 specific inhibition by S14 compound increases adult neurogenesis in the hippocampus and subventricular zone in rats probably via CREB pathway activation [209]. Together, these results show that it may be promising to target PDEs for neurogenesis activation in the context of PD and other neurodegenerative diseases. 

In that sense, we have previously shown that the three main events taking part in neurogenesis, proliferation, migration, and differentiation of neural stem cells through a neuronal phenotype, is enhanced in vitro when GSK-3 is inhibited. This inhibition, using tideglusib, a small molecule, promotes new neurons formation in the hippocampal dentate gyrus of adult rats [210]. In summary these results point to GSK-3 as a new promising target molecule to be used in the development of innovative treatments for neurodegenerative diseases, since its activity modulate the generation of new neurons and its integration in the hippocampus, representing new potential therapeutic drugs in neuroregenerative medicine.

Finally, the use of iPS cells through the somatic cell reprogramming approach is currently drawing attention from researchers because of their beneficial effects on neurodegenerative diseases like PD. For instance, a PD rat model with iPS cell-derived neuronal stem cells transplanted into the striatum showed improvement in functional defects of rotational asymmetry. In addition, those neuronal stem cells survived and integrated into the brains of transplanted PD rats and differentiated into neurons, including DA neurons [211]. Based on these results, clinical application of iPS cells for neurodegenerative diseases may be an attractive strategy in the near future.

### 4.7. Targeting Metal Ions Homeostasis Alteration

Nowadays, some of the developing drugs strategies to target metal ions homeostasis alteration in PD are the same than those used for AD. It has been shown that clioquinol or deferoxamine treatments improve the motor symptoms in PD models. Moreover, there is another disease-modifying drug against PD named deferiprone, which is a Fe chelator in phase 3 clinical trials. This compound is able to decrease the levels of iron deposits in the substantia nigra and the motor handicap progression in PD patients [66].

### 4.8. Other Targets

Mutations in *LRRK2* are the most common autosomal dominant cause of PD and appear to increase kinase activity. For this reason, an interesting target for PD treatment may be the inhibition of LRRK2 kinase activity, which provides neuroprotection in a variety of models [212]. Research efforts are being made to develop an effective and safe small-molecule inhibitor and, very recently, a phase 1 clinical trial started to evaluate the safety, tolerability, pharmacokinetics, and pharmacodynamics of the inhibitor DLN201 in PD patients (NCT03710707). Also, the use of LRRK2 antisense nucleotides is being considered [213].

## 5. Future Therapeutic Approaches in AD and PD

Here, we discuss some novel molecular targets which are in the pipeline and might be interesting for future therapeutic interventions in AD and PD. In the case of AD, genetic mutations in pathways associated with immune-inflammatory mediated responses, through differential expressions in microglial specific receptors (CD33, TREM2, and CR3) have been demonstrated to be strong risk factors for developing late onset AD (LOAD) [214,215,216]. Recently, a major microglial transmembrane signaling adaptor polypeptide, also called tyrosine kinase binding protein (TYROBP), which is a direct adaptor for TREM2, CD33, and CR3 activity and seems to be involved in AD, was identified. The function of TYROBP influences TREM2 activity; both factors and their dysregulation may be genetically related risks to AD. However, a deficiency of TYROBP in AD transgenic models was associated with a decrease in microglial activation around the plaques. Also, an alteration in microglial autophagy flux was observed and might be a possible novel therapeutic approach for AD. However, TYROBP deficiency also demonstrated a beneficiary role on other related proteinopathies of AD, like tau, synaptic integrity, and the lysosomal processes [214]. Furthermore, in PD, an alternative approach to stimulate autophagic flux might be targeting chaperone-mediated autophagy components, such as lysosome-associated membrane protein 2a (LAMP2a) and Hsc70. It has been observed that the overexpression of *LAMP2a* in vitro in the SH-SY5Y DA cell line and in rat primary cortical neurons and in vivo in nigral DA neurons decreases α-Syn aggregation and toxicity, thereby protecting these neurons [217].

Besides this, complement factor 3 (C3), which is a ligand for the complement C3 receptor (CR3), has been suggested as a possible factor in AD. C3 deficiency has been demonstrated to increase the Aβ plaque load while it is protective against age- and AD-related synaptic loss and cognitive decline by altering the glial response within the Aβ plaques. This suggests that the increase of fibrillar Aβ plaques is not likely to be the toxic species in AD. This increase of Aβ caused by C3 deficiency also suggests a decrease glial response as well as reduced Aβ phagocytosis [215,216]. Whether C3 deficiency has a protective role in Aβ toxicity or whether it causes the sequestration/aggregation of Aβ needs to be further investigated.

Both TYROBP deficiency and C3 deficiency need to be further explored to see how they protect or prevent dysregulation of these above-mentioned pathways to select the most promising multi-target drug candidate which might have a beneficiary influence on AD pathology.

Other receptor-mediated therapeutic tools are the toll-like receptor 5 (TLR5) and Nurr1, which both have demonstrated important value in idiopathic related pathways [218,219]. A recent study showed that soluble ectodomains of TLR5, which have a high binding affinity for oligomeric and fibrillary Aβ species, could be a novel immunotherapy against Aβ induced neurotoxicity [218]. Besides this, *Nurr1* is highly expressed in the glutamatergic neurons of the hippocampus and is dysregulated in AD and PD. Modulation with the Nurr1 agonist, amodiaquine, led to a reduction in Aβ plaque deposition and enhanced the effect of adult hippocampal neurogenesis, reducing neuronal loss and ameliorating microglia activation [219].

Both TYROBP deficiency and C3 deficiency, as well as the TLR5 and Nurr1 expression patterns, need to be further explored to see how they protect or prevent dysregulation of these above-mentioned pathways and to select the most promising multi-target drug candidate to encounter AD pathology.

Another neuroinflammatory therapeutic approach is targeting CCAAT/Enhancer Binding Protein β (C/EBPβ), a transcription factor that regulates a variety of processes in the brain. Among them, it has been described that this transcription factor regulates the expression of several genes implicated in inflammatory response and brain injury [220]. We recently showed that C/EBPβ silencing reduces glial activation and protects against neurodegeneration in a 6-OHDA rat model of PD [221]. However, targeting a transcription factor with so many regulated effectors can be challenging. For this reason, targeting neuroinflammation by the inhibition of specific C/EBPβ downstream effectors may be also a possible therapeutic approach for PD treatment.

## 6. Conclusions

Neurodegenerative diseases affect a large number of people nowadays and their incidence is increasing due to the aging population. So far, only symptomatic treatments are available. Thus, there is an urgent need to find novel therapeutic approaches to prevent or slow down the progression of these disorders. Here, we reviewed novel approaches to target altered pathways based on misfolding protein aggregation, mitochondrial dysfunction, oxidative stress, autophagy impairment, alterations in intracellular Ca^2+^ homeostasis, neuroinflammation, and neurogenesis impairment which are involved in these diseases.

Personalized medicine based on single or multi-target drug approaches, depending on the disease etiology (familial or idiopathic) and the disease progression in the patient, could be helpful for developing better understanding and finding disease modifying treatments that are cost-effective for the health care system. Besides this, promising data has been found regarding the early detection of effective target candidates and disease-modifying compounds that are currently in the pipeline. These data could bring us closer to a single or a combination of various therapeutic approaches to restore neurodegeneration, a feature of these diseases. 

## Supplementary Materials

Supplementary materials can be found at http://www.mdpi.com/1422-0067/20/3/719/s1.

## Abbreviations

4-HNE	4-hydroxynonenal
6-OHDA	6-hydroxydopamine
AChE	Acetylcholinesterase
AD	Alzheimer’s disease
ApoE4	Apolipoprotein-E4
Aβ	Amyloid beta
APP	Amyloid precursor protein
ATP13A2	ATPase cation transporting 13A2
BACE1	β-secretase
BBB	Blood–brain barrier
BDNF	Brain-derived neurotrophic factor
BPT	4-(1-benzylpiperidin-4-yl)thiosemicarbazones
C/EBPβ	CCAAT/Enhancer Binding Protein β
C3	Complement factor 3
CDNF	Cerebral dopamine neurotrophic factor
CNS	Central nervous system
CNTF	Ciliary neurotropic factor
CR3	Complement C3 receptor
CREB	cAMP response element binding protein
CRMP2	Collapsin response mediator protein 2
DA	Dopamigernic
DAMP	Damage-associated molecular pattern
DDQ	Diethyl(3,4-dihydroxyphenethylamino)(quinolin-4-l)methylphosphonate
E4FAD	APOE4-familial Alzheimer’s Disease
ECE-1	Endothelin converting enzyme 1
ECE-2	Endothelin converting enzyme 2
ER	Endoplasmic reticulum
ET-1	Endothelin-1 isopeptide
ETB	Endothelin B
FDA	Food and drug administration
GSK-3	Glycogen synthase kinase-3
GSTM1	Glutathione S transferases 1
IL-1β	Interleukin 1β
IL-10	Interleukin 10
KCC2A	Calcium/calmodulin-dependent protein kinase type II subunit α
KT2D	Kojo tacrines
LAMP2a	Lysosome-associated membrane protein 2a
LIF	Leukemia inhibitory factor
LKE	Lanthionine ketamine-ethyl ester
LOAD	Late onset Alzheimer’s Disease
LRRK2	Leucine-rich repeat kinase 2
LTD	Long-term depression
LTP	Long-term potentiation
MAM	Mitochondrial associated membranes
MANF	Mesencephalic astrocyte-derived neurotrophic factor
MCI	Mild cognitive impairment
MPAC	metal-protein-attenuating compounds
MPTP	1-methyl-4-phenyl-1,2,3,6-tetrahydropyridine
mtDNA	Mitochondrial DNA
NGF	Neural growth factor
NMDAR	*N*-methyl-d-aspartate receptor
NRP1	Neuropilin-1 receptor
Nurr1	Nuclear receptor related 1
PBPT	Pyridoxal 4-(1-benzylpiperidin-4-yl)thiosemicarbazone
pCRMP2	phosphorylated-CRPM2
PD	Parkinson’s disease
PGC1α	Coactivator 1-α
PINK1	PTEN induced kinase 1
PPARγ	Peroxisome proliferator-activated receptor-γ
PSEN-1	Presenilin-1
PSEN-2	Presenilin-2
ROS	Reactive oxygen species
sAPPα	Soluble amyloid precursor protein-α
sAPPβ	Soluble amyloid precursor protein-β
Sema3A	Semaphoring 3A
Sirt1	Sirtuin-1
Snag	Gamma soluble NSF attachment protein
SNpc	Substantia nigra pars compacta
α-Syn/SNCA	α-synuclein
TFEB	Transcription factor EB
TLR	Toll-like receptor
TNF-α	Tumor necrosis factor α
TREM2	Triggering receptor expressed on myeloid cells 2
TYROBP	Tyrosine kinase binding protein

## References

[1] GBD 2016 Parkinson’s Disease Collaborators (2018). Global, regional, and national burden of Parkinson’s disease, 1990–2016: A systematic analysis for the Global Burden of Disease Study 2016. Lancet Neurol..

[2] Prince M., Wimo A., Guerchet M., Ali G., Wu Y., Prina M. (2015). World Alzheimer Report 2015. The Global Impact of Dementia. Alzheimer’s Disease International.

[3] Scheltens P., Blennow K., Breteler M.M., de Strooper B., Frisoni G.B., Salloway S., Van der Flier W.M. (2016). Alzheimer’s disease. Lancet.

[4] O’Brien J.T., Thomas A. (2015). Vascular dementia. Lancet.

[5] Berg D., Postuma R.B., Bloem B., Chan P., Dubois B., Gasser T., Goetz C.G., Halliday G.M., Hardy J., Lang A.E. (2014). Time to redefine PD? Introductory statement of the MDS Task Force on the definition of Parkinson’s disease. Mov. Disord..

[6] Finkel S.I., Costa e Silva J., Cohen G., Miller S., Sartorius N. (1996). Behavioral and psychological signs and symptoms of dementia: A consensus statement on current knowledge and implications for research and treatment. Int. Psychogeriatr..

[7] Orsolini L., Tomasetti C., Valchera A., Vecchiotti R., Matarazzo I., Vellante F., Iasevoli F., Buonaguro E.F., Fornaro M., Fiengo A.L. (2016). An update of safety of clinically used atypical antipsychotics. Expert Opin. Drug Saf..

[8] Piersanti M., Capannolo M., Turchetti M., Serroni N., De Berardis D., Evangelista P., Costantini P., Orsini A., Rossi A., Maggio R. (2014). Increase in mortality rate in patients with dementia treated with atypical antipsychotics: A cohort study in outpatients in Central Italy. Riv. Psichiatr..

[9] Ostrowski S.M., Johnson K., Siefert M., Shank S., Sironi L., Wolozin B., Landreth G.E., Ziady A.G. (2016). Simvastatin inhibits protein isoprenylation in the brain. Neuroscience.

[10] Wolozin B., Wang S.W., Li N.C., Lee A., Lee T.A., Kazis L.E. (2007). Simvastatin is associated with a reduced incidence of dementia and Parkinson’s disease. BMC Med..

[11] Briggs R., Kennelly S.P., O’Neill D. (2016). Drug treatments in Alzheimer’s disease. Clin. Med..

[12] Oertel W., Schulz J.B. (2016). Current and experimental treatments of Parkinson disease: A guide for neuroscientists. J. Neurochem..

[13] Verstraeten A., Theuns J., Van Broeckhoven C. (2015). Progress in unraveling the genetic etiology of Parkinson disease in a genomic era. Trends Genet.

[14] Ransohoff R.M. (2016). How neuroinflammation contributes to neurodegeneration. Science.

[15] Jay T.R., von Saucken V.E., Landreth G.E. (2017). TREM2 in Neurodegenerative Diseases. Mol. Neurodegener..

[16] Bondi M.W., Edmonds E.C., Salmon D.P. (2017). Alzheimer’s Disease: Past, Present, and Future. J. Int. Neuropsychol. Soc..

[17] Michel P.P., Hirsch E.C., Hunot S. (2016). Understanding Dopaminergic Cell Death Pathways in Parkinson Disease. Neuron.

[18] Prentice H., Modi J.P., Wu J.Y. (2015). Mechanisms of Neuronal Protection against Excitotoxicity, Endoplasmic Reticulum Stress, and Mitochondrial Dysfunction in Stroke and Neurodegenerative Diseases. Oxid. Med. Cell. Longev..

[19] Winner B., Melrose H.L., Zhao C., Hinkle K.M., Yue M., Kent C., Braithwaite A.T., Ogholikhan S., Aigner R., Winkler J. (2011). Adult neurogenesis and neurite outgrowth are impaired in LRRK2 G2019S mice. Neurobiol. Dis..

[20] Wirths O. (2017). Altered neurogenesis in mouse models of Alzheimer disease. Neurogenesis.

[21] Ries H.M., Nussbaum-Krammer C. (2016). Shape matters: The complex relationship between aggregation and toxicity in protein-misfolding diseases. Essays Biochem..

[22] Glenner G.G., Wong C.W. (1984). Alzheimer’s disease and Down’s syndrome: Sharing of a unique cerebrovascular amyloid fibril protein. Biochem. Biophys. Res. Commun..

[23] Maccioni R.B., Farías G., Morales I., Navarrete L. (2010). The revitalized tau hypothesis on Alzheimer’s disease. Arch. Med. Res..

[24] Spillantini M.G., Schmidt M.L., Lee V.M., Trojanowski J.Q., Jakes R., Goedert M. (1997). Alpha-synuclein in Lewy bodies. Nature.

[25] Giasson B.I., Duda J.E., Murray I.V., Chen Q., Souza J.M., Hurtig H.I., Ischiropoulos H., Trojanowski J.Q., Lee V.M. (2000). Oxidative damage linked to neurodegeneration by selective alpha-synuclein nitration in synucleinopathy lesions. Science.

[26] Swerdlow R.H., Khan S.M. (2004). A “mitochondrial cascade hypothesis” for sporadic Alzheimer’s disease. Med. Hypotheses.

[27] Kozlov S., Afonin A., Evsyukov I., Bondarenko A. (2017). Alzheimer’s disease: As it was in the beginning. Rev. Neurosci..

[28] Arrasate M., Mitra S., Schweitzer E.S., Segal M.R., Finkbeiner S. (2004). Inclusion body formation reduces levels of mutant huntingtin and the risk of neuronal death. Nature.

[29] Van Giau V., An S.S.A., Hulme J.P. (2018). Mitochondrial therapeutic interventions in Alzheimer’s disease. J. Neurol. Sci..

[30] Fiorito V., Chiabrando D., Tolosano E. (2018). Mitochondrial Targeting in Neurodegeneration: A Heme Perspective. Pharmaceuticals.

[31] Briston T., Hicks A.R. (2018). Mitochondrial dysfunction and neurodegenerative proteinopathies: Mechanisms and prospects for therapeutic intervention. Biochem. Soc. Trans..

[32] Zaltieri M., Longhena F., Pizzi M., Missale C., Spano P., Bellucci A. (2015). Mitochondrial Dysfunction and α-Synuclein Synaptic Pathology in Parkinson’s Disease: Who’s on First?. Parkinsons Dis..

[33] Silva D.F., Esteves A.R., Oliveira C.R., Cardoso S.M. (2011). Mitochondria: The common upstream driver of amyloid-β and tau pathology in Alzheimer’s disease. Curr. Alzheimer Res..

[34] Truban D., Hou X., Caulfield T.R., Fiesel F.C., Springer W. (2017). PINK1, Parkin, and Mitochondrial Quality Control: What can we Learn about Parkinson’s Disease Pathobiology?. J. Parkinsons Dis..

[35] Narendra D., Tanaka A., Suen D.F., Youle R.J. (2008). Parkin is recruited selectively to impaired mitochondria and promotes their autophagy. J. Cell Biol..

[36] Palacino J.J., Sagi D., Goldberg M.S., Krauss S., Motz C., Wacker M., Klose J., Shen J. (2004). Mitochondrial dysfunction and oxidative damage in parkin-deficient mice. J. Biol. Chem..

[37] Gautier C.A., Kitada T., Shen J. (2008). Loss of PINK1 causes mitochondrial functional defects and increased sensitivity to oxidative stress. Proc. Natl. Acad. Sci. USA.

[38] Taira T., Saito Y., Niki T., Iguchi-Ariga S.M., Takahashi K., Ariga H. (2004). DJ-1 has a role in antioxidative stress to prevent cell death. EMBO Rep..

[39] Kim R.H., Smith P.D., Aleyasin H., Hayley S., Mount M.P., Pownall S., Wakeham A., You-Ten A.J., Kalia S.K., Horne P. (2005). Hypersensitivity of DJ-1-deficient mice to 1-methyl-4-phenyl-1,2,3,6-tetrahydropyrindine (MPTP) and oxidative stress. Proc. Natl. Acad. Sci. USA.

[40] Shim J.H., Yoon S.H., Kim K.H., Han J.Y., Ha J.Y., Hyun D.H., Paek S.H., Kang U.J., Zhuang X., Son J.H. (2011). The antioxidant Trolox helps recovery from the familial Parkinson’s disease-specific mitochondrial deficits caused by PINK1- and DJ-1-deficiency in dopaminergic neuronal cells. Mitochondrion.

[41] Sarasija S., Norman K.R. (2018). Role of Presenilin in Mitochondrial Oxidative Stress and Neurodegeneration in *Caenorhabditis elegans*. Antioxidants.

[42] Kim G.H., Kim J.E., Rhie S.J., Yoon S. (2015). The Role of Oxidative Stress in Neurodegenerative Diseases. Exp. Neurobiol..

[43] Shelat P.B., Chalimoniuk M., Wang J.H., Strosznajder J.B., Lee J.C., Sun A.Y., Simonyi A., Sun G.Y. (2008). Amyloid beta peptide and NMDA induce ROS from NADPH oxidase and AA release from cytosolic phospholipase A2 in cortical neurons. J. Neurochem..

[44] Atwood C.S., Moir R.D., Huang X., Scarpa R.C., Bacarra N.M., Romano D.M., Hartshorn M.A., Tanzi R.E., Bush A.I. (1998). Dramatic aggregation of Alzheimer Aβ by Cu(II) is induced by conditions representing physiological acidosis. J. Biol. Chem..

[45] Garcia-Ruiz P.J., Espay A.J. (2017). Parkinson Disease: An Evolutionary Perspective. Front. Neurol..

[46] Guzman J.N., Sánchez-Padilla J., Chan C.S., Surmeier D.J. (2009). Robust pacemaking in substantia nigra dopaminergic neurons. J. Neurosci..

[47] Pacelli C., Giguère N., Bourque M.J., Lévesque M., Slack R.S., Trudeau L. (2015). Elevated Mitochondrial Bioenergetics and Axonal Arborization Size Are Key Contributors to the Vulnerability of Dopamine Neurons. Curr. Biol..

[48] Cheignon C., Tomas M., Bonnefont-Rousselot D., Faller P., Hureau C., Collin F. (2018). Oxidative stress and the amyloid beta peptide in Alzheimer’s disease. Redox Biol..

[49] Puspita L., Chung S.Y., Shim J.W. (2017). Oxidative stress and cellular pathologies in Parkinson’s disease. Mol. Brain.

[50] Radi E., Formichi P., Battisti C., Federico A. (2014). Apoptosis and oxidative stress in neurodegenerative diseases. J. Alzheimers Dis..

[51] Karabiyik C., Lee M.J., Rubinsztein D.C. (2017). Autophagy impairment in Parkinson’s disease. Essays Biochem..

[52] Ciechanover A., Brundin P. (2003). The ubiquitin proteasome system in neurodegenerative diseases: Sometimes the chicken, sometimes the egg. Neuron.

[53] Menzies F.M., Fleming A., Caricasole A., Bento C.F., Andrews S.P., Ashkenazi A., Füllgrabe J., Jackson A., Jimenez Sanchez M., Karabiyik C. (2017). Autophagy and Neurodegeneration: Pathogenic Mechanisms and Therapeutic Opportunities. Neuron.

[54] Giordano S., Darley-Usmar V., Zhang J. (2014). Autophagy as an essential cellular antioxidant pathway in neurodegenerative disease. Redox Biol..

[55] Lionaki E., Markaki M., Palikaras K., Tavernarakis N. (2015). Mitochondria, autophagy and age-associated neurodegenerative diseases: New insights into a complex interplay. Biochim. Biophys. Acta.

[56] Pchitskaya E., Popugaeva E., Bezprozvanny I. (2018). Calcium signaling and molecular mechanisms underlying neurodegenerative diseases. Cell Calcium.

[57] Surmeier D.J., Halliday G.M., Simuni T. (2017). Calcium, mitochondrial dysfunction and slowing the progression of Parkinson’s disease. Exp. Neurol..

[58] Rannikko E.H., Weber S.S., Kahle P.J. (2015). Exogenous α-synuclein induces toll-like receptor 4 dependent inflammatory responses in astrocytes. BMC Neurosci..

[59] Amor S., Peferoen L.A., Vogel D.Y., Breur M., van der Valk P., Baker D., van Noort J.M. (2014). Inflammation in neurodegenerative diseases—An update. Immunology.

[60] Winner B., Winkler J. (2015). Adult neurogenesis in neurodegenerative diseases. Cold Spring Harb. Perspect. Biol..

[61] Ghosal K., Stathopoulos A., Pimplikar S.W. (2010). APP intracellular domain impairs adult neurogenesis in transgenic mice by inducing neuroinflammation. PLoS ONE.

[62] Le Grand J.N., Gonzalez-Cano L., Pavlou M.A., Schwamborn J.C. (2015). Neural stem cells in Parkinson’s disease: A role for neurogenesis defects in onset and progression. Cell Mol. Life Sci..

[63] Naumann N., Alpár A., Ueberham U., Arendt T., Gärtner U. (2010). Transgenic expression of human wild-type amyloid precursor protein decreases neurogenesis in the adult hippocampus. Hippocampus.

[64] Peng J., Andersen J.K. (2011). Mutant α-synuclein and aging reduce neurogenesis in the acute 1-methyl-4-phenyl-1,2,3,6-tetrahydropyridine model of Parkinson’s disease. Aging Cell.

[65] Barnham K.J., Masters C.L., Bush A.I. (2004). Neurodegenerative diseases and oxidative stress. Nat. Rev. Drug Dis..

[66] Belaidi A.A., Bush A.I. (2016). Iron neurochemistry in Alzheimer’s disease and Parkinson’s disease: Targets for therapeutics. J. Neurochem..

[67] Stelmashook E.V., Isaev N.K., Genrikhs E.E., Amelkina G.A., Khaspekov L.G., Skrebitsky V.G., Illarioshkin S.N. (2014). Role of zinc and copper ions in the pathogenetic mechanisms of Alzheimer’s and Parkinson’s diseases. Biochemistry.

[68] Parsons C.G., Danysz W., Dekundy A., Pulte I. (2013). Memantine and cholinesterase inhibitors: Complementary mechanisms in the treatment of Alzheimer’s disease. Neurotox Res..

[69] Gulati A., Hornick M.G., Briyal S., Lavhale M.S. (2018). A novel neuroregenerative approach using ET(B) receptor agonist, IRL-1620, to treat CNS disorders. Physiol. Res..

[70] Watkins P.B., Zimmerman H.J., Knapp M.J., Gracon S.I., Lewis K.W. (1994). Hepatotoxic effects of tacrine administration in patients with Alzheimer’s disease. JAMA.

[71] van Dyck C.H. (2018). Anti-Amyloid-β Monoclonal Antibodies for Alzheimer’s Disease: Pitfalls and Promise. Biol. Psychiatry.

[72] Penninkilampi R., Brothers H.M., Eslick G.D. (2017). Safety and Efficacy of Anti-Amyloid-β Immunotherapy in Alzheimer’s Disease: A Systematic Review and Meta-Analysis. J. Neuroimmune Pharmacol..

[73] Adolfsson O., Pihlgren M., Toni N., Varisco Y., Buccarello A.L., Antoniello K., Lohmann S., Piorkowska K., Gafner V., Atwal J.K. (2012). An effector-reduced anti-β-amyloid (Aβ) antibody with unique Aβ binding properties promotes neuroprotection and glial engulfment of Aβ. J. Neurosci..

[74] Palanimuthu D., Poon R., Sahni S., Anjum R., Hibbs D., Lin H.Y., Bernhardt P.V., Kalinowski D.S., Richardson D.R. (2017). A novel class of thiosemicarbazones show multi-functional activity for the treatment of Alzheimer’s disease. Eur. J. Med. Chem..

[75] Grasso G.I., Bellia F., Arena G., Satriano C., Vecchio G., Rizzarelli E. (2017). Multitarget trehalose-carnosine conjugates inhibit Aβ aggregation, tune copper(II) activity and decrease acrolein toxicity. Eur. J. Med. Chem..

[76] Dgachi Y., Martin H., Malek R., Jun D., Janockova J., Sepsova V., Soukup O., Iriepa I., Moraleda I., Maalej E. (2019). Synthesis and biological assessment of KojoTacrines as new agents for Alzheimer’s disease therapy. J. Enzyme Inhib. Med. Chem..

[77] Campagna J., Spilman P., Jagodzinska B., Bai D., Hatami A., Zhu C., Bilousova T., Jun M., Elias C.J., Pham J. (2018). A small molecule ApoE4-targeted therapeutic candidate that normalizes sirtuin 1 levels and improves cognition in an Alzheimer’s disease mouse model. Sci. Rep..

[78] Theendakara V., Peters-Libeu C.A., Spilman P., Poksay K.S., Bredesen D.E., Rao R.V. (2016). Direct Transcriptional Effects of Apolipoprotein E. J. Neurosci..

[79] Min S.W., Sohn P.D., Li Y., Devidze N., Johnson J.R., Krogan N.J., Masliah E., Mok S.A., Gestwicki J.E., Gan L. (2018). SIRT1 Deacetylates Tau and Reduces Pathogenic Tau Spread in a Mouse Model of Tauopathy. J. Neurosci..

[80] Bredesen D.E. (2009). Neurodegeneration in Alzheimer’s disease: Caspases and synaptic element interdependence. Mol. Neurodegener..

[81] Chen J., Zhou Y., Mueller-Steiner S., Chen L.F., Kwon H., Yi S., Mucke L., Gan L. (2005). SIRT1 protects against microglia-dependent amyloid-β toxicity through inhibiting NF-kappaB signaling. J. Biol. Chem..

[82] Bai X., Yao L., Ma X., Xu X. (2018). Small Molecules as SIRT Modulators. Mini Rev. Med. Chem..

[83] Villalba J.M., Alcaín F.J. (2012). Sirtuin activators and inhibitors. Biofactors.

[84] Alcaín F.J., Villalba J.M. (2009). Sirtuin activators. Expert. Opin. Ther. Pat..

[85] Gulati A., Kumar A., Shahani B.T. (1996). Cardiovascular effects of centrally administered endothelin-1 and its relationship to changes in cerebral blood flow. Life Sci..

[86] Gulati A., Kumar A., Morrison S., Shahani B.T. (1997). Effect of centrally administered endothelin agonists on systemic and regional blood circulation in the rat: Role of sympathetic nervous system. Neuropeptides.

[87] Palmer J.C., Baig S., Kehoe P.G., Love S. (2009). Endothelin-converting enzyme-2 is increased in Alzheimer’s disease and up-regulated by Aβ. Am. J. Pathol..

[88] Palmer J.C., Barker R., Kehoe P.G., Love S. (2012). Endothelin-1 is elevated in Alzheimer’s disease and upregulated by amyloid-β. J. Alzheimers Dis..

[89] Palmer J.C., Tayler H.M., Love S. (2013). Endothelin-converting enzyme-1 activity, endothelin-1 production, and free radical-dependent vasoconstriction in Alzheimer’s disease. J. Alzheimers Dis..

[90] Wang D.S., Dickson D.W., Malter J.S. (2006). β-Amyloid degradation and Alzheimer’s disease. J. Biomed. Biotechnol..

[91] Briyal S., Shepard C., Gulati A. (2014). Endothelin receptor type B agonist, IRL-1620, prevents beta amyloid (Aβ) induced oxidative stress and cognitive impairment in normal and diabetic rats. Pharmacol. Biochem. Behav..

[92] Briyal S., Nguyen C., Leonard M., Gulati A. (2015). Stimulation of endothelin B receptors by IRL-1620 decreases the progression of Alzheimer’s disease. Neuroscience.

[93] Pozhilenkova E.A., Lopatina O.L., Komleva Y.K., Salmin V.V., Salmina A.B. (2017). Blood-brain barrier-supported neurogenesis in healthy and diseased brain. Rev. Neurosci..

[94] Goetzl E.J., Kapogiannis D., Schwartz J.B., Lobach I.V., Goetzl L., Abner E.L., Jicha G.A., Karydas A.M., Boxer A., Miller B.L. (2016). Decreased synaptic proteins in neuronal exosomes of frontotemporal dementia and Alzheimer’s disease. FASEB J..

[95] Kuruva C.S., Manczak M., Yin X., Ogunmokun G., Reddy A.P., Reddy P.H. (2017). Aqua-soluble DDQ reduces the levels of Drp1 and Aβ and inhibits abnormal interactions between Aβ and Drp1 and protects Alzheimer’s disease neurons from Aβ- and Drp1-induced mitochondrial and synaptic toxicities. Hum. Mol. Genet..

[96] Hensley K., Kursula P. (2016). Collapsin Response Mediator Protein-2 (CRMP2) is a Plausible Etiological Factor and Potential Therapeutic Target in Alzheimer’s Disease: Comparison and Contrast with Microtubule-Associated Protein Tau. J. Alzheimers Dis..

[97] Goshima Y., Nakamura F., Strittmatter P., Strittmatter S.M. (1995). Collapsin-induced growth cone collapse mediated by an intracellular protein related to UNC-33. Nature.

[98] Kawano Y., Yoshimura T., Tsuboi D., Kawabata S., Kaneko-Kawano T., Shirataki H., Takenawa T., Kaibuchi K. (2005). CRMP-2 is involved in kinesin-1-dependent transport of the Sra-1/WAVE1 complex and axon formation. Mol. Cell Biol..

[99] Harris-White M.E., Ferbas K.G., Johnson M.F., Eslami P., Poteshkina A., Venkova K., Christov A., Hensley K. (2015). A cell-penetrating ester of the neural metabolite lanthionine ketimine stimulates autophagy through the mTORC1 pathway: Evidence for a mechanism of action with pharmacological implications for neurodegenerative pathologies. Neurobiol. Dis..

[100] Caccamo A., De Pinto V., Messina A., Branca C., Oddo S. (2014). Genetic reduction of mammalian target of rapamycin ameliorates Alzheimer’s disease-like cognitive and pathological deficits by restoring hippocampal gene expression signature. J. Neurosci..

[101] Kodama Y., Murakumo Y., Ichihara M., Kawai K., Shimono Y., Takahashi M. (2004). Induction of CRMP-2 by GDNF and analysis of the CRMP-2 promoter region. Biochem. Biophys. Res. Commun..

[102] Chu C.C., Wang J.J., Chen K.T., Shieh J.P., Wang L.K., Shui H.A., Ho S.T. (2010). Neurotrophic effects of tianeptine on hippocampal neurons: A proteomic approach. J. Proteome Res..

[103] Caberlotto L., Carboni L., Zanderigo F., Andreetta F., Andreoli M., Gentile G., Razzoli M. (2013). Differential effects of glycogen synthase kinase 3 (GSK3) inhibition by lithium or selective inhibitors in the central nervous system. Naunyn Schmiedebergs Arch. Pharmacol..

[104] Zhang X., Hernandez I., Rei D., Mair W., Laha J.K., Cornwell M.E., Cuny G.D., Tsai L.H., Steen J.A., Kosik K.S. (2013). Diaminothiazoles modify Tau phosphorylation and improve the tauopathy in mouse models. J. Biol. Chem..

[105] Cole A.R., Knebel A., Morrice N.A., Robertson L.A., Irving A.J., Connolly C.N., Sutherland C. (2004). GSK-3 phosphorylation of the Alzheimer epitope within collapsin response mediator proteins regulates axon elongation in primary neurons. J. Biol. Chem..

[106] Lin P.C., Chan P.M., Hall C., Manser E. (2011). Collapsin response mediator proteins (CRMPs) are a new class of microtubule-associated protein (MAP) that selectively interacts with assembled microtubules via a taxol-sensitive binding interaction. J. Biol. Chem..

[107] Cole A.R., Noble W., van Aalten L., Plattner F., Meimaridou R., Hogan D., Taylor M., LaFrancois J., Gunn-Moore F., Verkhratsky A. (2007). Collapsin response mediator protein-2 hyperphosphorylation is an early event in Alzheimer’s disease progression. J. Neurosci..

[108] Williamson R., van Aalten L., Mann D.M., Platt B., Plattner F., Bedford L., Mayer J., Howlett D., Usardi A., Sutherland C. (2011). CRMP2 hyperphosphorylation is characteristic of Alzheimer’s disease and not a feature common to other neurodegenerative diseases. J. Alzheimers Dis..

[109] Amani M., Shokouhi G., Salari A.A. (2018). Minocycline prevents the development of depression-like behavior and hippocampal inflammation in a rat model of Alzheimer’s disease. Psychopharmacology.

[110] Baazaoui N., Iqbal K. (2018). A Novel Therapeutic Approach to Treat Alzheimer’s Disease by Neurotrophic Support During the Period of Synaptic Compensation. J. Alzheimers Dis..

[111] Du J., Liang Y., Xu F., Sun B., Wang Z. (2013). Trehalose rescues Alzheimer’s disease phenotypes in APP/PS1 transgenic mice. J. Pharm. Pharmacol..

[112] Portbury S.D., Hare D.J., Sgambelloni C., Perronnes K., Portbury A.J., Finkelstein D.I., Adlard P.A. (2017). Trehalose Improves Cognition in the Transgenic Tg2576 Mouse Model of Alzheimer’s Disease. J. Alzheimers Dis..

[113] Minami S.S., Min S.W., Krabbe G., Wang C., Zhou Y., Asgarov R., Li Y., Martens L.H., Elia L.P., Ward M.E. (2014). Progranulin protects against amyloid β deposition and toxicity in Alzheimer’s disease mouse models. Nat. Med..

[114] Toh H., Chitramuthu B.P., Bennett H.P., Bateman A. (2011). Structure, function, and mechanism of progranulin; the brain and beyond. J. Mol. Neurosci..

[115] Arsenijevic Y., Weiss S., Schneider B., Aebischer P. (2001). Insulin-like growth factor-I is necessary for neural stem cell proliferation and demonstrates distinct actions of epidermal growth factor and fibroblast growth factor-2. J. Neurosci..

[116] Ahmed S., Mahmood Z., Javed A., Hashmi S.N., Zerr I., Zafar S., Zahid S. (2017). Effect of Metformin on Adult Hippocampal Neurogenesis: Comparison with Donepezil and Links to Cognition. J. Mol. Neurosci..

[117] Ayton S., Lei P., Bush A.I. (2015). Biometals and their therapeutic implications in Alzheimer’s disease. Neurotherapeutics.

[118] Schaeffer V., Meyer L., Patte-Mensah C., Eckert A., Mensah-Nyagan A.G. (2008). Dose-dependent and sequence-sensitive effects of amyloid-β peptide on neurosteroidogenesis in human neuroblastoma cells. Neurochem. Int..

[119] Schaeffer V., Patte-Mensah C., Eckert A., Mensah-Nyagan A.G. (2006). Modulation of neurosteroid production in human neuroblastoma cells by Alzheimer’s disease key proteins. J. Neurobiol..

[120] Mensah-Nyagan A.G., Do-Rego J.L., Beaujean D., Luu-The V., Pelletier G., Vaudry H. (1999). Neurosteroids: Expression of steroidogenic enzymes and regulation of steroid biosynthesis in the central nervous system. Pharmacol. Rev..

[121] Patte-Mensah C., Kibaly C., Mensah-Nyagan A.G. (2005). Substance P inhibits progesterone conversion to neuroactive metabolites in spinal sensory circuit: A potential component of nociception. Proc. Natl. Acad. Sci. USA.

[122] Lejri I., Grimm A., Miesch M., Geoffroy P., Eckert A., Mensah-Nyagan A.G. (2017). Allopregnanolone and its analog BR 297 rescue neuronal cells from oxidative stress-induced death through bioenergetic improvement. Biochim. Biophys. Acta Mol. Basis Dis..

[123] Chen G.H., Wang H., Yang Q.G., Tao F., Wang C., Xu D.X. (2011). Acceleration of age-related learning and memory decline in middle-aged CD-1 mice due to maternal exposure to lipopolysaccharide during late pregnancy. Behav. Brain Res..

[124] Chen S., Wang J.M., Irwin R.W., Yao J., Liu L., Brinton R.D. (2011). Allopregnanolone promotes regeneration and reduces β-amyloid burden in a preclinical model of Alzheimer’s disease. PLoS ONE.

[125] Wang J.M., Johnston P.B., Ball B.G., Brinton R.D. (2005). The neurosteroid allopregnanolone promotes proliferation of rodent and human neural progenitor cells and regulates cell-cycle gene and protein expression. J. Neurosci..

[126] Wang J.M., Singh C., Liu L., Irwin R.W., Chen S., Chung E.J., Thompson R.F., Brinton R.D. (2010). Allopregnanolone reverses neurogenic and cognitive deficits in mouse model of Alzheimer’s disease. Proc. Natl. Acad. Sci. USA.

[127] Lewis J., Melrose H., Bumcrot D., Hope A., Zehr C., Lincoln S., Braithwaite A., He Z., Ogholikhan S., Hinkle K. (2008). In vivo silencing of alpha-synuclein using naked siRNA. Mol. Neurodegener..

[128] Cooper J.M., Wiklander P.B., Nordin J.Z., Al-Shawi R., Wood M.J., Vithlani M., Schapira A.H., Simons J.P., El-Andaloussi S., Alvarez-Erviti L. (2014). Systemic exosomal siRNA delivery reduced alpha-synuclein aggregates in brains of transgenic mice. Mov. Disord..

[129] Wong Y.C., Krainc D. (2017). α-synuclein toxicity in neurodegeneration: Mechanism and therapeutic strategies. Nat. Med..

[130] Ellis J.M., Fell M.J. (2017). Current approaches to the treatment of Parkinson’s Disease. Bioorg. Med. Chem. Lett..

[131] Li W., West N., Colla E., Pletnikova O., Troncoso J.C., Marsh L., Dawson T.M., Jäkälä P., Hartmann T., Price D.L., Lee M.K. (2005). Aggregation promoting C-terminal truncation of alpha-synuclein is a normal cellular process and is enhanced by the familial Parkinson’s disease-linked mutations. Proc. Natl. Acad. Sci. USA.

[132] Wang W., Nguyen L.T., Burlak C., Chegini F., Guo F., Chataway T., Ju S., Fisher O.S., Miller D.W., Datta D. (2016). Caspase-1 causes truncation and aggregation of the Parkinson’s disease-associated protein α-synuclein. Proc. Natl. Acad. Sci. USA.

[133] Bassil F., Fernagut P.O., Bezard E., Pruvost A., Leste-Lasserre T., Hoang Q.Q., Ringe D., Petsko G.A., Meissner W.G. (2016). Reducing C-terminal truncation mitigates synucleinopathy and neurodegeneration in a transgenic model of multiple system atrophy. Proc. Natl. Acad. Sci. USA.

[134] Wagner J., Ryazanov S., Leonov A., Levin J., Shi S., Schmidt F., Prix C., Pan-Montojo F., Bertsch U., Mitteregger-Kretzschmar G. (2013). Anle138b: A novel oligomer modulator for disease-modifying therapy of neurodegenerative diseases such as prion and Parkinson’s disease. Acta Neuropathol..

[135] Pujols J., Peña-Díaz S., Lázaro D.F., Peccati F., Pinheiro F., González D., Carija A., Navarro S., Conde-Giménez M., García J. (2018). Small molecule inhibits α-synuclein aggregation, disrupts amyloid fibrils, and prevents degeneration of dopaminergic neurons. Proc. Natl. Acad. Sci. USA.

[136] Mahul-Mellier A.L., Fauvet B., Gysbers A., Dikiy I., Oueslati A., Georgeon S., Lamontanara A.J., Bisquertt A., Eliezer D., Masliah E. (2014). c-Abl phosphorylates α-synuclein and regulates its degradation: Implication for α-synuclein clearance and contribution to the pathogenesis of Parkinson’s disease. Hum. Mol. Genet..

[137] Games D., Valera E., Spencer B., Rockenstein E., Mante M., Adame A., Patrick C., Ubhi K., Nuber S., Sacayon P. (2014). Reducing C-terminal-truncated alpha-synuclein by immunotherapy attenuates neurodegeneration and propagation in Parkinson’s disease-like models. J. Neurosci..

[138] Tran H.T., Chung C.H.-Y., Iba M., Zhang B., Trojanowski J.Q., Luk K.C., Lee V.M.J. (2014). α-Synuclein immunotherapy blocks uptake and templated propagation of misfolded α-synuclein and neurodegeneration. Cell Rep..

[139] Loria F., Vargas J.Y., Bousset L., Syan S., Salles A., Melki R., Zurzolo C. (2017). α-Synuclein transfer between neurons and astrocytes indicates that astrocytes play a role in degradation rather than in spreading. Acta Neuropathol..

[140] Wang B., Underwood R., Kamath A., Britain C., McFerrin M.B., McLean P.J., Volpicelli-Daley L.A., Whitaker R.H., Placzek W.J., Becker K. (2018). 14-3-3 Proteins Reduce Cell-to-Cell Transfer and Propagation of Pathogenic α-Synuclein. J. Neurosci..

[141] Koentjoro B., Park J.S., Sue C.M. (2017). Nix restores mitophagy and mitochondrial function to protect against PINK1/Parkin-related Parkinson’s disease. Sci. Rep..

[142] Bingol B., Tea J.S., Phu L., Reichelt M., Bakalarski C.E., Song Q., Foreman O., Kirkpatrick D.S., Sheng M. (2014). The mitochondrial deubiquitinase USP30 opposes parkin-mediated mitophagy. Nature.

[143] Wang Y., Serricchio M., Jauregui M., Shanbhag R., Stoltz T., Di Paolo C.T., Kim P.K., McQuibban G.A. (2015). Deubiquitinating enzymes regulate PARK2-mediated mitophagy. Autophagy.

[144] Cornelissen T., Haddad D., Wauters F., Van Humbeeck C., Mandemakers W., Koentjoro B., Sue C., Gevaert K., De Strooper B., Verstreken P. (2014). The deubiquitinase USP15 antagonizes Parkin-mediated mitochondrial ubiquitination and mitophagy. Hum. Mol. Genet..

[145] Durcan T.M., Tang M.Y., Pérusse J.R., Dashti E.A., Aguileta M.A., McLelland G.L., Gros P., Shaler T.A., Faubert D., Coulombe B., Fon E.A. (2014). USP8 regulates mitophagy by removing K6-linked ubiquitin conjugates from parkin. EMBO J..

[146] Nakamura N., Hirose S. (2008). Regulation of mitochondrial morphology by USP30, a deubiquitinating enzyme present in the mitochondrial outer membrane. Mol. Biol. Cell.

[147] Thobois S. (2015). USP30: A new promising target for Parkinson’s disease?. Mov. Disord..

[148] Hayashi G., Jasoliya M., Sahdeo S., Saccà F., Pane C., Filla A., Marsili A., Puorro G., Lanzillo R., Brescia Morra V., Cortopassi G. (2017). Dimethyl fumarate mediates Nrf2-dependent mitochondrial biogenesis in mice and humans. Hum. Mol. Genet..

[149] Kaidery N.A., Banerjee R., Yang L., Smirnova N.A., Hushpulian D.M., Liby K.T., Williams C.R., Yamamoto M., Kensler T.W., Ratan R.R. (2013). Targeting Nrf2-mediated gene transcription by extremely potent synthetic triterpenoids attenuate dopaminergic neurotoxicity in the MPTP mouse model of Parkinson’s disease. Antioxid. Redox Signal..

[150] Houten S.M., Auwerx J. (2004). PGC-1alpha: Turbocharging mitochondria. Cell.

[151] Li X., Wang H., Gao Y., Li L., Tang C., Wen G., Yang Y., Zhuang Z., Zhou M., Mao L. (2016). Quercetin induces mitochondrial biogenesis in experimental traumatic brain injury via the PGC-1α signaling pathway. Am. J. Transl. Res..

[152] Choong C.J., Mochizuki H. (2017). Gene therapy targeting mitochondrial pathway in Parkinson’s disease. J. Neural. Transm..

[153] Jin H., Kanthasamy A., Ghosh A., Anantharam V., Kalyanaraman B., Kanthasamy A.G. (2014). Mitochondria-targeted antioxidants for treatment of Parkinson’s disease: Preclinical and clinical outcomes. Biochim. Biophys. Acta.

[154] Beal M.F., Oakes D., Shoulson I., Henchcliffe C., Galpern W.R., Haas R., Juncos J.L., Nutt J.G., Voss T.S., Ravina B. (2014). A randomized clinical trial of high-dosage coenzyme Q10 in early Parkinson disease: No evidence of benefit. JAMA Neurol..

[155] Kieburtz K., Tilley B.C., Elm J.J., Babcock D., Hauser R., Ross G.W., Augustine A.H., Augustine E.U., Aminoff M.J., Bodis-Wollner I.G. (2015). Effect of creatine monohydrate on clinical progression in patients with Parkinson disease: A randomized clinical trial. JAMA.

[156] Ghosh A., Chandran K., Kalivendi S.V., Joseph J., Antholine W.E., Hillard C.J., Kanthasamy A., Kalyanaraman B. (2010). Neuroprotection by a mitochondria-targeted drug in a Parkinson’s disease model. Free Radic. Biol. Med..

[157] McManus M.J., Murphy M.P., Franklin J.L. (2011). The mitochondria-targeted antioxidant MitoQ prevents loss of spatial memory retention and early neuropathology in a transgenic mouse model of Alzheimer’s disease. J. Neurosci..

[158] Snow B.J., Rolfe F.L., Lockhart M.M., Frampton C.M., O’Sullivan J.D., Fung V., Smith R.A., Murphy M.P., Taylor K.M., Group P.S. (2010). A double-blind, placebo-controlled study to assess the mitochondria-targeted antioxidant MitoQ as a disease-modifying therapy in Parkinson’s disease. Mov. Disord..

[159] Rivero-Ríos P., Madero-Pérez J., Fernández B., Hilfiker S. (2016). Targeting the Autophagy/Lysosomal Degradation Pathway in Parkinson’s Disease. Curr. Neuropharmacol..

[160] Settembre C., Di Malta C., Polito V.A., Garcia Arencibia M., Vetrini F., Erdin S., Erdin S.U., Huynh T., Medina D., Colella P. (2011). TFEB links autophagy to lysosomal biogenesis. Science.

[161] Awad O., Sarkar C., Panicker L.M., Miller D., Zeng X., Sgambato J.A., Lipinski M.M., Feldman R.A. (2015). Altered TFEB-mediated lysosomal biogenesis in Gaucher disease iPSC-derived neuronal cells. Hum. Mol. Genet..

[162] Decressac M., Mattsson B., Weikop P., Lundblad M., Jakobsson J., Björklund A. (2013). TFEB-mediated autophagy rescues midbrain dopamine neurons from α-synuclein toxicity. Proc. Natl. Acad. Sci. USA.

[163] Kilpatrick K., Zeng Y., Hancock T., Segatori L. (2015). Genetic and chemical activation of TFEB mediates clearance of aggregated α-synuclein. PLoS ONE.

[164] Siddiqui A., Bhaumik D., Chinta S.J., Rane A., Rajagopalan S., Lieu C.A., Lithgow G.J., Andersen J.K. (2015). Mitochondrial Quality Control via the PGC1α-TFEB Signaling Pathway Is Compromised by Parkin Q311X Mutation But Independently Restored by Rapamycin. J. Neurosci..

[165] Moors T.E., Hoozemans J.J., Ingrassia A., Beccari T., Parnetti L., Chartier-Harlin M.C., van de Berg W.D. (2017). Therapeutic potential of autophagy-enhancing agents in Parkinson’s disease. Mol. Neurodegener..

[166] Spencer B., Potkar R., Trejo M., Rockenstein E., Patrick C., Gindi R., Adame A., Wyss-Coray T., Masliah E. (2009). Beclin 1 gene transfer activates autophagy and ameliorates the neurodegenerative pathology in alpha-synuclein models of Parkinson’s and Lewy body diseases. J. Neurosci..

[167] Wang K., Huang J., Xie W., Huang L., Zhong C., Chen Z. (2016). Beclin1 and HMGB1 ameliorate the α-synuclein-mediated autophagy inhibition in PC12 cells. Diagn. Pathol..

[168] Savolainen M.H., Richie C.T., Harvey B.K., Männistö P.T., Maguire-Zeiss K.A., Myöhänen T.T. (2014). The beneficial effect of a prolyl oligopeptidase inhibitor, KYP-2047, on alpha-synuclein clearance and autophagy in A30P transgenic mouse. Neurobiol. Dis..

[169] Baltazar G.C., Guha S., Lu W., Lim J., Boesze-Battaglia K., Laties A.M., Tyagi P., Kompella U.B., Mitchell C.H. (2012). Acidic nanoparticles are trafficked to lysosomes and restore an acidic lysosomal pH and degradative function to compromised ARPE-19 cells. PLoS ONE.

[170] Schapira A.H., Gegg M.E. (2013). Glucocerebrosidase in the pathogenesis and treatment of Parkinson disease. Proc. Natl. Acad. Sci. USA.

[171] Aflaki E., Borger D.K., Moaven N., Stubblefield B.K., Rogers S.A., Patnaik S., Schoenen F.J., Westbroek W., Zheng W., Sullivan P. (2016). A New Glucocerebrosidase Chaperone Reduces α-Synuclein and Glycolipid Levels in iPSC-Derived Dopaminergic Neurons from Patients with Gaucher Disease and Parkinsonism. J. Neurosci..

[172] Baehrecke E.H. (2005). Autophagy: Dual roles in life and death?. Nat. Rev. Mol. Cell Biol..

[173] Chan C.S., Guzman J.N., Ilijic E., Mercer J.N., Rick C., Tkatch T., Meredith G.E., Surmeier D.J. (2007). ‘Rejuvenation’ protects neurons in mouse models of Parkinson’s disease. Nature.

[174] Salthun-Lassalle B., Hirsch E.C., Wolfart J., Ruberg M., Michel P.P. (2004). Rescue of mesencephalic dopaminergic neurons in culture by low-level stimulation of voltage-gated sodium channels. J. Neurosci..

[175] Guzman J.N., Sanchez-Padilla J., Wokosin D., Kondapalli J., Ilijic E., Schumacker P.T., Surmeier D.J. (2010). Oxidant stress evoked by pacemaking in dopaminergic neurons is attenuated by DJ-1. Nature.

[176] McGeer P.L., Itagaki S., Boyes B.E., McGeer E.G. (1988). Reactive microglia are positive for HLA-DR in the substantia nigra of Parkinson’s and Alzheimer’s disease brains. Neurology.

[177] Gerhard A., Pavese N., Hotton G., Turkheimer F., Es M., Hammers A., Eggert K., Oertel W., Banati R.B., Brooks D.J. (2006). In vivo imaging of microglial activation with [11C](R)-PK11195 PET in idiopathic Parkinson’s disease. Neurobiol. Dis..

[178] Ouchi Y., Yagi S., Yokokura M., Sakamoto M. (2009). Neuroinflammation in the living brain of Parkinson’s disease. Parkinsonism Relat. Disord..

[179] Martinez B., Peplow P.V. (2018). Neuroprotection by immunomodulatory agents in animal models of Parkinson’s disease. Neural. Regen. Res..

[180] Lee F.S., Rajagopal R., Kim A.H., Chang P.C., Chao M.V. (2002). Activation of Trk neurotrophin receptor signaling by pituitary adenylate cyclase-activating polypeptides. J. Biol. Chem..

[181] Delgado M., Leceta J., Ganea D. (2003). Vasoactive intestinal peptide and pituitary adenylate cyclase-activating polypeptide inhibit the production of inflammatory mediators by activated microglia. J. Leukoc. Biol..

[182] Mäkelä J., Koivuniemi R., Korhonen L., Lindholm D. (2010). Interferon-gamma produced by microglia and the neuropeptide PACAP have opposite effects on the viability of neural progenitor cells. PLoS ONE.

[183] Reglodi D., Kiss P., Lubics A., Tamas A. (2011). Review on the protective effects of PACAP in models of neurodegenerative diseases in vitro and in vivo. Curr. Pharm. Des..

[184] Lee E.H., Seo S.R. (2014). Neuroprotective roles of pituitary adenylate cyclase-activating polypeptide in neurodegenerative diseases. BMB Rep..

[185] Morales-Garcia J.A., Redondo M., Alonso-Gil S., Gil C., Perez C., Martinez A., Santos A., Perez-Castillo A. (2011). Phosphodiesterase 7 inhibition preserves dopaminergic neurons in cellular and rodent models of Parkinson disease. PLoS ONE.

[186] Perez-Gonzalez R., Pascual C., Antequera D., Bolos M., Redondo M., Perez D.I., Pérez-Grijalba V., Krzyzanowska A., Sarasa M., Gil C. (2013). Phosphodiesterase 7 inhibitor reduced cognitive impairment and pathological hallmarks in a mouse model of Alzheimer’s disease. Neurobiol. Aging.

[187] Mestre L., Redondo M., Carrillo-Salinas F.J., Morales-García J.A., Alonso-Gil S., Pérez-Castillo A., Gil C., Martínez A., Guaza C. (2015). PDE7 inhibitor TC3.6 ameliorates symptomatology in a model of primary progressive multiple sclerosis. Br. J. Pharmacol..

[188] Morales-Garcia J.A., Aguilar-Morante D., Hernandez-Encinas E., Alonso-Gil S., Gil C., Martinez A., Santos A., Perez-Castillo A. (2015). Silencing phosphodiesterase 7B gene by lentiviral-shRNA interference attenuates neurodegeneration and motor deficits in hemiparkinsonian mice. Neurobiol. Aging.

[189] Aviles-Olmos I., Limousin P., Lees A., Foltynie T. (2013). Parkinson’s disease, insulin resistance and novel agents of neuroprotection. Brain.

[190] Carta A.R., Frau L., Pisanu A., Wardas J., Spiga S., Carboni E. (2011). Rosiglitazone decreases peroxisome proliferator receptor-γ levels in microglia and inhibits TNF-α production: New evidences on neuroprotection in a progressive Parkinson’s disease model. Neuroscience.

[191] Swanson C.R., Joers V., Bondarenko V., Brunner K., Simmons H.A., Ziegler T.E., Kemnitz J.W., Johnson J.A., Emborg M.E. (2011). The PPAR-γ agonist pioglitazone modulates inflammation and induces neuroprotection in parkinsonian monkeys. J. Neuroinflamm..

[192] Brauer R., Bhaskaran K., Chaturvedi N., Dexter D.T., Smeeth L., Douglas I. (2015). Glitazone Treatment and Incidence of Parkinson’s Disease among People with Diabetes: A Retrospective Cohort Study. PLoS Med..

[193] NINDS Exploratory Trials in Parkinson Disease (NET-PD) FS-ZONE Investigators (2015). Pioglitazone in early Parkinson’s disease: A phase 2, multicentre, double-blind, randomised trial. Lancet Neurol..

[194] Bonato J.M., Bassani T.B., Milani H., Vital M.A.B.F., de Oliveira R.M.W. (2018). Pioglitazone reduces mortality, prevents depressive-like behavior, and impacts hippocampal neurogenesis in the 6-OHDA model of Parkinson’s disease in rats. Exp. Neurol..

[195] Hughes P.E., Alexi T., Walton M., Williams C.E., Dragunow M., Clark R.G., Gluckman P.D. (1999). Activity and injury-dependent expression of inducible transcription factors, growth factors and apoptosis-related genes within the central nervous system. Prog. Neurobiol..

[196] Ibáñez C.F., Andressoo J.O. (2017). Biology of GDNF and its receptors - Relevance for disorders of the central nervous system. Neurobiol. Dis..

[197] Rangasamy S.B., Soderstrom K., Bakay R.A., Kordower J.H. (2010). Neurotrophic factor therapy for Parkinson’s disease. Prog. Brain Res..

[198] Vilar M., Mira H. (2016). Regulation of Neurogenesis by Neurotrophins during Adulthood: Expected and Unexpected Roles. Front. Neurosci..

[199] Francardo V., Schmitz Y., Sulzer D., Cenci M.A. (2017). Neuroprotection and neurorestoration as experimental therapeutics for Parkinson’s disease. Exp. Neurol..

[200] Lindahl M., Saarma M., Lindholm P. (2017). Unconventional neurotrophic factors CDNF and MANF: Structure, physiological functions and therapeutic potential. Neurobiol. Dis..

[201] Lindholm P., Voutilainen M.H., Laurén J., Peränen J., Leppänen V.M., Andressoo J.O., Lindahl M., Janhunen S., Kalkkinen N., Timmusk T. (2007). Novel neurotrophic factor CDNF protects and rescues midbrain dopamine neurons in vivo. Nature.

[202] Voutilainen M.H., Bäck S., Pörsti E., Toppinen L., Lindgren L., Lindholm P., Peränen J., Saarma M., Tuominen R.K. (2009). Mesencephalic astrocyte-derived neurotrophic factor is neurorestorative in rat model of Parkinson’s disease. J. Neurosci..

[203] Heldin C.H., Westermark B. (1999). Mechanism of action and in vivo role of platelet-derived growth factor. Physiol. Rev..

[204] Pietz K., Odin P., Funa K., Lindvall O. (1996). Protective effect of platelet-derived growth factor against 6-hydroxydopamine-induced lesion of rat dopaminergic neurons in culture. Neurosci. Lett..

[205] Zachrisson O., Zhao M., Andersson A., Dannaeus K., Häggblad J., Isacson R., Nielsen E., Patrone C., Rönnholm H., Wikstrom L., Delfani K., McCormack A.L. (2011). Restorative effects of platelet derived growth factor-BB in rodent models of Parkinson’s disease. J. Parkinsons Dis..

[206] Paul G., Zachrisson O., Varrone A., Almqvist P., Jerling M., Lind G., Rehncrona S., Linderoth B., Bjartmarz H., Shafer L.L. (2015). Safety and tolerability of intracerebroventricular PDGF-BB in Parkinson’s disease patients. J. Clin. Investig..

[207] Kim C.H., Han B.S., Moon J., Kim D.J., Shin J., Rajan S., Nguyen Q.T., Sohn M., Kim W.G., Han M. (2015). Nuclear receptor Nurr1 agonists enhance its dual functions and improve behavioral deficits in an animal model of Parkinson’s disease. Proc. Natl. Acad. Sci. USA.

[208] Winner B., Regensburger M., Schreglmann S., Boyer L., Prots I., Rockenstein E., Mante M., Zhao C., Winkler J., Masliah E. (2012). Role of α-synuclein in adult neurogenesis and neuronal maturation in the dentate gyrus. J. Neurosci..

[209] Morales-Garcia J.A., Echeverry-Alzate V., Alonso-Gil S., Sanz-SanCristobal M., Lopez-Moreno J.A., Gil C., Martinez A., Santos A., Perez-Castillo A. (2017). Phosphodiesterase7 Inhibition Activates Adult Neurogenesis in Hippocampus and Subventricular Zone In Vitro and In Vivo. Stem Cells.

[210] Morales-Garcia J.A., Luna-Medina R., Alonso-Gil S., Sanz-SanCristobal M., Palomo V., Gil C., Santos A., Martinez A., Perez-Castillo A. (2012). Glycogen synthase kinase 3 inhibition promotes adult hippocampal neurogenesis in vitro and in vivo. ACS Chem. Neurosci..

[211] Han F., Wang W., Chen B., Chen C., Li S., Lu X., Duan J., Zhang Y., Zhang Y.A., Guo W. (2015). Human induced pluripotent stem cell-derived neurons improve motor asymmetry in a 6-hydroxydopamine-induced rat model of Parkinson’s disease. Cytotherapy.

[212] West A.B. (2017). Achieving neuroprotection with LRRK2 kinase inhibitors in Parkinson disease. Exp. Neurol..

[213] Zhao H.T., John N., Delic V., Ikeda-Lee K., Kim A., Weihofen A., Swayze E.E., Kordasiewicz H.B., West A.B., Volpicelli-Daley L.A. (2017). LRRK2 Antisense Oligonucleotides Ameliorate α-Synuclein Inclusion Formation in a Parkinson’s Disease Mouse Model. Mol. Ther. Nucleic Acids.

[214] Haure-Mirande J.V., Audrain M., Fanutza T., Kim S.H., Klein W.L., Glabe C., Readhead B., Dudley J.T., Blitzer R.D., Wang M. (2017). Deficiency of TYROBP, an adapter protein for TREM2 and CR3 receptors, is neuroprotective in a mouse model of early Alzheimer’s pathology. Acta Neuropathol..

[215] Hernandez-Encinas E., Aguilar-Morante D., Morales-Garcia J.A., Gine E., Sanz-SanCristobal M., Santos A., Perez-Castillo A. (2016). Complement component 3 (C3) expression in the hippocampus after excitotoxic injury: Role of C/EBPβ. J. Neuroinflamm..

[216] Shi Q., Chowdhury S., Ma R., Le K.X., Hong S., Caldarone B.J., Stevens B., Lemere C.A. (2017). Complement C3 deficiency protects against neurodegeneration in aged plaque-rich APP/PS1 mice. Sci. Transl. Med..

[217] Xilouri M., Brekk O.R., Landeck N., Pitychoutis P.M., Papasilekas T., Papadopoulou-Daifoti Z., Kirik D., Stefanis L. (2013). Boosting chaperone-mediated autophagy in vivo mitigates α-synuclein-induced neurodegeneration. Brain.

[218] Chakrabarty P., Li A., Ladd T.B., Strickland M.R., Koller E.J., Burgess J.D., Funk C.C., Cruz P.E., Allen M., Yaroshenko M. (2018). TLR5 decoy receptor as a novel anti-amyloid therapeutic for Alzheimer’s disease. J. Exp. Med..

[219] Moon M., Jung E.S., Jeon S.G., Cha M.Y., Jang Y., Kim W., Lopes C., Mook-Jung I., Kim K.S. (2018). Nurr1 (NR4A2) regulates Alzheimer’s disease-related pathogenesis and cognitive function in the 5XFAD mouse model. Aging Cell.

[220] Hernandez-Encinas E., Aguilar-Morante D., Cortes-Canteli M., Morales-Garcia J.A., Gine E., Santos A., Perez-Castillo A. (2015). CCAAT/enhancer binding protein β directly regulates the expression of the complement component 3 gene in neural cells: Implications for the pro-inflammatory effects of this transcription factor. J. Neuroinflamm..

[221] Morales-Garcia J.A., Gine E., Hernandez-Encinas E., Aguilar-Morante D., Sierra-Magro A., Sanz-SanCristobal M., Alonso-Gil S., Sanchez-Lanzas R., Castaño J.G., Santos A. (2017). CCAAT/Enhancer binding protein β silencing mitigates glial activation and neurodegeneration in a rat model of Parkinson’s disease. Sci. Rep..

