# Supplementary material for: Novel Approaches for the Treatment of Alzheimer’s and Parkinson’s Disease
Source: Int J Mol Sci. 2019 Feb 8;20(3):719. doi: 10.3390/ijms20030719 (PMC6386829; doi:10.3390/ijms20030719)
Supplement: Supplementary file 1 [file ijms-20-00719-s001.pdf]

Table S1. Influence of novel drugs targets for treatment of Alzheimer disease.

Targets	Class Type	Compounds	Direct and indirect effects	Publications/Clinical Trials
**Amyloid-Beta and excitotoxicity**	AChE inhibitors	Donepezil, Galantamine, Rivastigmine	Improvement in memory and cognitive and impairment as well as behaviour impairment	68
		Tacrine derivates (KT2D)	Neuroprotection against Aβ	70, 75
	NMDAR antagonist	Memantine	↓ Excitotoxicity, improvement in memory and cognitive impairment	68
		Sirt1 enhancer (A03)	↑ Sirt1 expression and memory improvement	78, 79, 80, 81, 82, 83, 84
	Immunotherapy (IMT)	Gantenerumab	Passive Immunization by ↓ Aβ spreading and aggregation, ↑ microglial activation and ↑ phagocytosis	71, 72 NCT03443973, NCT03444870, NCT02051608, NCT01224106, NCT01760005
		Crenezumab	Passive Immunization by ↓ Aβ spreading and aggregation, ↑ microglial activation and ↑ phagocytosis	71, 72, 73, NCT02353598, NCT01998841, NCT02670083, NCT03443973, NCT03491150),
		Aducanumab	Passive Immunization by ↓ Aβ spreading and aggregation, ↑ microglial activation and ↑ phagocytosis	71,72, NCT01677572, NCT02484547, NCT02477800, NCT03639987
	ETB receptor agonist	IRL-1620	↑ Clearance of ET-1 and Aβ, ↓ oxidative stress, ↑ neurogenesis and synaptogenesis improve memory deficiency	69, 91, 92, 93, 94
**Mitochondria**	Dopamine derived structure	DDQ	↑ Mitochondrial biogenesis, ↑ Mitochondrial fusion activity, ↓ mitochondrial fission activity, ↓ Aβ toxicity	95
**Autophagy**	CRMP2 modulator	LKE	Normalization of CRMP2 phosphorylation, ↓ Aβ-plaques load, ↓ phosphorylated tau, ↑ growth factor-dependent neurite outgrowth as well as autophagy related processes	99, 100
	Cyclin-dependent kinase modulator	Cdk5 inhibitors	↑ Autophagy by ↓ CRMP2 hyper-phosphorylation	103, 104
**Neuroinflammation**	Tetracycline antibiotic	Minocycline	↓ Proinflammatory (TNF-α and IL-1β)and ↑ anti-inflammatory cytokines (IL-10)	109
**Neurogenesis and neuronal survival**	Ciliary Neurotophic factor	P021	Inhibition of LIF signalling and ↑ the BDNF expression; ↑ neurogenesis by rescuing dendritic, synaptic and cognitive impairment; ↓ Aβ and tau mediated aggregation	110
	Natural disaccharide	Trehalose	↓ Aβ aggregation, ↑ hippocampal neurogenesis and synaptic plasticity, ↑ neuronal growth and survival	75, 111, 112, 113, 114
	Type II anti-diabetic	Metformin	↑ neurogenesis, ↓ oxidative stress, ↓ cognitive impairment	68, 116
	Allopregnanolones	BR297	↑ steroidogenesis, improving mitochondrial bioenergetics by ↑ cellular ATP production and ↓ oxidative stress, Preventing neuronal cell death	120, 121, 122
	Nurr1 activators	amodiaquine	↑ Dopaminergic neurogenesis, ↑ hippocampal neurogenesis ↓ Aβ plaques deposition, ↓ neuronal loss & microglia activation	207, 219
**Metal ions homeostasis**	Cu^{2+}, Zn^{2+}, Fe^{3+} chelators	PBT1 and PBT2	↓ Aβ-plaques load and ↓ cognitive impairment	65, 66, 117
	AChE inhibitor	BPT derivatives	Inhibition of Cu^{2+}- and Fe^{3+}-mediated Aβ aggregation	74, 75
	Fe2+ chelators	DFO	↓ α-Syn- and Aβ-mediated-iron load, ↓ cognitive impairment	117

Arrows indicates; (↑), increase and (↓), decrease. All abbreviation are explained in text.

Table S2. Influence of novel drugs targets for treatment of Parkinson's disease.

Targets	Compounds	Direct and indirect effects	Publications/Clinical Trials
**α-Syn and LRRK2**	Antisense oligonucleotides	↓ α-Syn synthesis and ↓ LRRK2 synthesis	129, 213
	DLN201	Inhibition of LRRK2 kinase activity	NCT03710707
	GZ/SAR40261	GCS inhibitor, ↑ α-Syn degradation and ↑ lysosomal activity	130, NCT02906020
	Ambroxol	GBA activator, ↑ α-Syn degradation, ↑ lysosomal activity	NCT02941822
	Caspase-1 inhibitor	↓ C-terminal truncation and ↓ α-Syn cleavage and toxicity	132, 133
	Anle138 and SynuClean-D	Stabilization of α-Syn structure and prevention oligomerization by ↓ α-Syn misfolding and aggregation	134
	NTP200-11		NCT02906020
	Nilotinib	Inhibition of c-Abl kinase activity, ↓ α-Syn aggregation	136, NCT03205488
	PRX002 and BIIB054	Passive Immunization by ↓ α-Syn spreading	NCT02157714, NCT03318523
	PD01A and PD03A	Active Immunization by ↓ α-Syn spreading	NCT02216188, NCT02267434
**Mitochondria**	STI-571	↑ Parkin-mediated mitophagy	144
	NIX	Mitochondrial autophagy receptor Nip3-like protein X restores mitophagy	140
	USP30 inhibitors	↑ Parkin-mediated mitophagy	147
	BG12	↑ Mitochondrial biogenesis by activation of Nrf2	148
	Quercetin	↑ Mitochondrial biogenesis by activation of PGC1α	150, 151
**Autophagy**	TFEB	↑ Autophagy	160, 162, 163
	KYP-2047	↑ Beclin-1 mediated autophagy by inhibition of PREP	168
	PLGA-aNPs	↑ Autophagy by ↑ lysosomal activity	169
	NCGC607	↑ Autophagy by ↑ chaperone-GCase mediated lysosomal activity	165, 170, 171
**Ca^{2+} homeostasis**	Israpidine	Inhibition of Ca^{2+} channels, ↓ mitochondrial oxidative stress	173, 175, NCT02168842
**Neuroinflammation**	Maxadilan	↑ PACAP activity by cAMP, ↓ microglial pro-inflammatory cytokines	180, 181, 182
	S14	PDE7 inhibition, ↑ adult neurogenesis and ↑ anti-inflammatory responses	185, 186, 187, 209
	Glitazones derivatives	↑ PPARγ activation, ↑ neuroprotection, ↓ microglial pro-inflammatory cytokines	189, 190, 191, 192, 193
**Neurogenesis and neuronal survival**	CDNF and MANF	Protection and repair of dopaminergic neurons	202, NCT03775538
	PDGF-BB	↑ Dopaminergic neurogenesis in the subventricular zone	205, 206
	Nurr1 activators	↑ Dopaminergic neurogenesis, ↑ hippocampal neurogenesis<br>↓ Aβ plaques deposition, ↓ neuronal loss & microglia activation	207, 219
	CREB activator (Rolipram)	↑ Dopamine neurogenesis by ↑ of dendritic outgrowth	208
	Tideglusib	↑ Neurogenesis in the dentate gyrus of the hippocampus by inhibition of GSK-3	210
	IPS cells transplantation	↑ Dopaminergic cells	211
**Metal ions homeostasis**	PBT1 and PBT2	Cu^{2+}, Zn^{2+}, Fe^{3+} chelators; ↓ Aβ-plaques load and ↓ cognitive impairment	65, 66, 117
	DFO and DFP	Fe^{2+} chelators, ↓ α-Syn- and Aβ-mediated-iron load, ↓ motor & cognitive impairment	117

Arrows indicates; (↑), increase and (↓), decrease. All abbreviations are explained in text.
